# Genistein: An Integrative Overview of Its Mode of Action, Pharmacological Properties, and Health Benefits

**DOI:** 10.1155/2021/3268136

**Published:** 2021-07-19

**Authors:** Javad Sharifi-Rad, Cristina Quispe, Muhammad Imran, Abdur Rauf, Muhammad Nadeem, Tanweer Aslam Gondal, Bashir Ahmad, Muhammad Atif, Mohammad S. Mubarak, Oksana Sytar, Oxana Mihailovna Zhilina, Ekaterina Robertovna Garsiya, Antonella Smeriglio, Domenico Trombetta, Daniel Gabriel Pons, Miquel Martorell, Susana M. Cardoso, Ahmad Faizal Abdull Razis, Usman Sunusi, Ramla Muhammad Kamal, Lia Sanda Rotariu, Monica Butnariu, Anca Oana Docea, Daniela Calina

**Affiliations:** ^1^Phytochemistry Research Center, Shahid Beheshti University of Medical Sciences, Tehran, Iran; ^2^Facultad de Ciencias de la Salud, Universidad Arturo Prat, Avda. Arturo Prat 2120, Iquique 1110939, Chile; ^3^University Institute of Diet and Nutritional Sciences, Faculty of Allied Health Sciences, The University of Lahore, Lahore, Pakistan; ^4^Department of Chemistry, University of Swabi, Anbar-, 23561 Khyber Pakhtunkhwa, Pakistan; ^5^Department of Environmental Sciences, COMSATS Institute of Information Technology, Vehari-, Pakistan; ^6^School of Exercise and Nutrition, Deakin University, Victoria 3125, Australia; ^7^Center of Biotechnology and Microbiology, University of Peshawar, Peshawar-, 25120 KPK, Pakistan; ^8^Department of Clinical Laboratory Sciences, College of Applied Medical Sciences, Jouf University, Sakaka 72341, Saudi Arabia; ^9^Department of Chemistry, The University of Jordan, Amman 11942, Jordan; ^10^Department of Plant Biology Department, Institute of Biology, Taras Shevchenko National University of Kyiv, Volodymyrska Str., 64, Kyiv 01033, Ukraine; ^11^Department of Plant Physiology, Slovak University of Agriculture, A. Hlinku 2, 94976 Nitra, Slovakia; ^12^Department of Organic Chemistry, Pyatigorsk Medical-Pharmaceutical Institute (PMPI), Branch of Volgograd State Medical University, Ministry of Health of Russia, Pyatigorsk 357532, Russia; ^13^Department of Pharmacognosy, Botany and Technology of Phytopreparations, Pyatigorsk Medical-Pharmaceutical Institute (PMPI), Branch of Volgograd State Medical University, Ministry of Health of Russia, Pyatigorsk 357532, Russia; ^14^Department of Chemical, Biological, Pharmaceutical and Environmental Sciences, University of Messina, Italy; ^15^Grupo Multidisciplinar de Oncología Traslacional (GMOT), Institut Universitari d'Investigació en Ciències de la Salut (IUNICS), Universitat de les Illes Balears (UIB), Instituto de Investigación Sanitaria Illes Balears (IdISBa), Palma 07122, Spain; ^16^Department of Nutrition and Dietetics, Faculty of Pharmacy, University of Concepción, Concepción 4070386, Chile; ^17^Unidad de Desarrollo Tecnológico, Universidad de Concepción UDT, Concepción 4070386, Chile; ^18^LAQV-REQUIMTE, Department of Chemistry, University of Aveiro, 3810-193 Aveiro, Portugal; ^19^Department of Food Science, Faculty of Food Science and Technology, Universiti Putra Malaysia, 43400, UPM Serdang, Selangor, Malaysia; ^20^Natural Medicines and Products Research Laboratory, Institute of Bioscience, Universiti Putra Malaysia, 43400 UPM Serdang, Selangor, Malaysia; ^21^Department of Biochemistry, Bayero University Kano, PMB 3011 Kano, Nigeria; ^22^Department of Pharmacology, Federal University Dutse, PMB 7156 Dutse Jigawa State, Nigeria; ^23^Banat's University of Agricultural Sciences and Veterinary Medicine “King Michael I of Romania” from Timisoara, Romania; ^24^Department of Toxicology, University of Medicine and Pharmacy of Craiova, 200349 Craiova, Romania; ^25^Department of Clinical Pharmacy, University of Medicine and Pharmacy of Craiova, 200349 Craiova, Romania

## Abstract

Genistein is an isoflavone first isolated from the brooming plant Dyer's *Genista tinctoria* L. and is widely distributed in the Fabaceae family. As an isoflavone, mammalian genistein exerts estrogen-like functions. Several biological effects of genistein have been reported in preclinical studies, such as the antioxidant, anti-inflammatory, antibacterial, and antiviral activities, the effects of angiogenesis and estrogen, and the pharmacological activities on diabetes and lipid metabolism. The purpose of this review is to provide up-to-date evidence of preclinical pharmacological activities with mechanisms of action, bioavailability, and clinical evidence of genistein. The literature was researched using the most important keyword “genistein” from the PubMed, Science, and Google Scholar databases, and the taxonomy was validated using The Plant List. Data were also collected from specialized books and other online resources. The main positive effects of genistein refer to the protection against cardiovascular diseases and to the decrease of the incidence of some types of cancer, especially breast cancer. Although the mechanism of protection against cancer involves several aspects of genistein metabolism, the researchers attribute this effect to the similarity between the structure of soy genistein and that of estrogen. This structural similarity allows genistein to displace estrogen from cellular receptors, thus blocking their hormonal activity. The pharmacological activities resulting from the experimental studies of this review support the traditional uses of genistein, but in the future, further investigations are needed on the efficacy, safety, and use of nanotechnologies to increase bioavailability and therapeutic efficacy.

## 1. Introduction

Nowadays, due to the increase in life expectancy, one of the main goals of scientific research is to counteract the onset of age-related diseases. Although it is well known that genetics plays a key role, it is also proven that lifestyle, therefore dietary habits as well as physical activity, plays a fundamental role in the onset of these pathologies [1, 2]. From this point of view, recently, functional foods as well as the nutraceutical field attract a growing interest [3, 4]. In particular, the latter leads to the development of products based on plant extract and/or their isolated bioactive compounds with well-recognized and, over time, always more in-depth investigated biological properties [5, 6]. Genistein has been originally identified in *Genista tinctoria* L., from which its name is derived; genistein is widely distributed in leguminous plant foods as well as in seeds, fruits, and vegetables such as alfalfa and clover sprouts, broccoli, cauliflower, sunflower, barley meal, caraway, and clover seeds [7].

Soybean, a cholesterol-free and high-protein legume, is the major source of genistein.

Originally from Asia, soy is part of the legume species, its grains growing in pods. In food, we use only berries, but in industry and medicine, we use other parts of the plant, including the root. It contains essential amino acids and, almost 40%, proteins, lipids, carbohydrates, mineral salts, enzymes, lecithins, and vitamins A, B_1_, B_2_, C, D, and E. The soybean plant grows to a medium height, has leaves colored in intense green, and has a small flower, white or purple. Originally launched in eastern China, soybean crops have spread rapidly throughout the planet, mainly due to the high nutritional value of the grains [8].

Genistein is generally attained through plant secondary metabolites and leguminous plants [9, 10] fulfilling various roles, for instance, UV filtration, plant pigmentation, and symbiotic nitrogen fixation. It has been shown that certain foods are poor or lacking, for example, soy oil and soy sauce, while other ones such as soybeans, soy nuts, soy powder, soy milk, and tofu contain a variable amount of genistein (1.9-18.5 *μ*g/g). However, the most genistein-rich foods are those fermented (miso and natto), which contain 38.5-230 *μ*g/g of genistein, due to the *β*-glycosyl bond cleavage of genistin (7-*O*-*β*-D-glucoside form of genistein, naturally occurring in plants) by microbes during the fermentation process [11].

**Figure 1:**
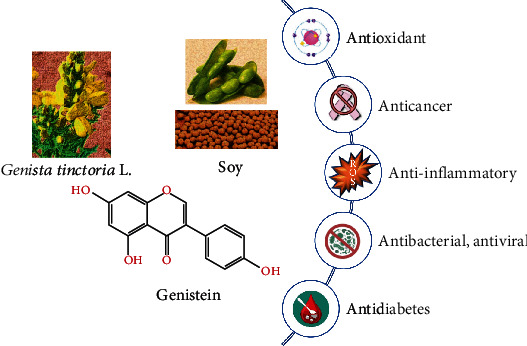
Importance of genistein for therapeutic purposes.

The recognition of the different beneficial effects of isoflavones in recent years, such as the relief of menopausal symptoms and breast and prostate cancers and the incidence of cardiovascular disease, osteoporosis, obesity and diabetes, cognitive functions, and virus infections, has greatly increased the market for soy-based products (Figure 1).

Recently various studies have concluded that genistein can help treat or prevent osteoporosis and heart diseases [9, 12, 13]. The main problem is the great variability regarding the isoflavone content among soy-based foods, not only between the different brands but also between the different lots of the same brand [14]. Furthermore, the introduction of different soy or purified isoflavone-based nutraceuticals has even more magnified the problem. In light of this, the use of standardized extracts, as well as a more controlled and consistent labeling, is advisable.

This review is aimed at providing updated evidence of preclinical pharmacological activities, bioavailability, and clinical evidence of genistein. It emphasized the clinical trials involving genistein into antioxidant, anticancer, cytotoxic, and anti-inflammatory activities, climacteric symptoms, and therapeutic effects on diabetes, lipid metabolism, depression, neurodegeneration, bone health, and cardiovascular disease.

## 2. Review Methodology

The current review was conducted by researching and collecting the most relevant literature from the scientific databases PubMed, Science, and Google Scholar. The search terms were “genistein,” “bioavailability,” “pharmacology,” “molecule mechanisms,” and “clinical studies.” The selected articles were evaluated in detail for a proper evaluation. Therefore, *in vitro* and *in vivo* experimental pharmacological studies on compounds and plant extracts, types of preclinical experiments, and doses and concentrations at which pharmacological properties, mechanisms, and molecular targets of genistein action were demonstrated were evaluated. Also, the most relevant clinical trials were included. The chemical structures were validated with PubChem and SciFinder. The scientific name of the plants was made according to The Plant List (https://www.theplantlist.org).

## 3. Chemical Structure and Bioavailability of Genistein

**Figure 2:**
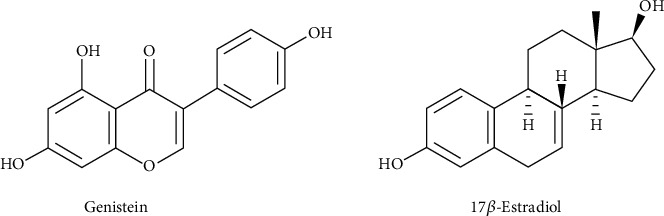
Genistein and 17*β*-estradiol chemical structures.

Genistein, one of the most known and investigated isoflavones, belongs to the group of aglycones. Isoflavones are present almost exclusively in glycosylated forms in natural sources and, only after food processing, become available in the biologically active forms, the aglycones [15]. In mammalians, isoflavones exert estrogen-like functions. In particular, they may act as estrogen agonists, showing synergic function with endogenous hormones (estradiol, E2, or 17*β*-estradiol), or as estrogen antagonists, blocking the estrogenic receptors (ER *α* and *β*) or inducing a conformational change of the same lead to its functional property loss [14]. The estrogen-like activity of genistein (5,7-dihydroxy-3-(4-hydroxyphenyl)chromen-4-one) is due in particular to carbons 4 and 7 on the phenol rings, which are similar in structure and functionality to the phenol groups on E2 (Figure 2), making it able to bind equally to both the isoforms *α* and *β* of the estrogen receptor (ER) [16, 17].

However, it is well known that the endocrine effects of genistein are also attributable to its main metabolite, also common to other isoflavones, the (−)-(S)-equol, a competent phytoestrogen generated by intestinal microbiota metabolism [17].

There are two types of estimations for the genistein oral bioavailability that were discussed in available literature data. One is absolute bioavailability which is calculated by comparing the plasma/urine AUC_oral_ to AUC_iv_ after dose correction (classic pharmacokinetic definition) [18]. The other types of estimations for the genistein oral bioavailability are to count urine or plasma AUC_oral_ and receive the % recovery established on the administrated dose. It is generally used in nutrition or clinical pharmacokinetic studies as intravenous administration is not accessible due to ethical or practical topics [19].

Furthermore, many pharmacokinetic investigations showed low oral bioavailability of genistein. Its tissue or plasma concentrations have been much lower than its *in vitro* IC_50_ [20] which may influence its *in vivo* efficiency. The low oral bioavailability is a disputed topic for developing genistein as a chemoprevention agent because of unclear therapeutic effects of genistein and broad interindividual diversity in clinical studies.

At the same time, bioavailability studies on portal vein plasma levels showed that the bioavailability of genistein is greater for the aglycon than for its glycoside. Genistein is partially absorbed in its glycosidic form [21]. Genistin, a glycosidic form of genistein, is mostly present in soy-derived foods. At the same time, another study showed that the oral bioavailability of genistein is greater compared to that of genistin [22].

Nowadays, studies of genistein bioavailability are developing intending to improve it. It was confirmed that Pluronic F127 polymeric micelles can increase the oral bioavailability of simple water-soluble genistein [23]. The nanoprecipitation technique using Eudragit® E100 as carriers and an optimized formulation of the mass ratio (genistein : Eudragit E100, 1 : 10) were used to prepare genistein nanoparticles which were effectively used for the efficient delivery of poorly water-soluble drugs by oral administration [24]. Genistein in combination with carbon-14 ([^14^C] genistein) showed absolute bioavailability in the rats with some differences in male and female rats [25]. At the same time, the systemic bioavailability and maximum serum concentration of [^13^C] genistein were significantly higher compared to that of [^13^C] daidzein in an experiment with premenopausal women [26]. Enhanced bioavailability of genistein by complexation with *β*-cyclodextrin in rats has been observed [27]. The starch-genistein complexes increase genistein bioavailability [28].

In a study with bioavailability of pure isoflavones in healthy humans and analysis of commercial soy isoflavone supplements, differences were found in the pharmacokinetics of isoflavone glycosides compared with their respective beta-glycosides. The apparent volume of distribution of isoflavones confirms extensive tissue distribution after absorption. The systemic bioavailability of genistein was estimated to be much greater than that of another isoflavone daidzein [20]. No significant genistein metabolism and bioavailability in the intestinal epithelial Caco-2 cells appeared whereas the glycosides were mainly metabolized to their respective aglycones [29].

## 4. Preclinical Pharmacological Activities of Genistein

### 4.1. Antioxidant Activity

In the last two decades, the appearance of degenerative processes associated with chronic diseases is correlated in molecular biology with the existence of harmful excess of free radicals, promoters of oxidative processes harmful to the body [30, 31]. Antioxidants are always at the forefront of the body's effective defense against free radicals [32, 33]. The need for a cell to survive depends very much on its oxygenation, but the presence of oxygen can lead to the oxidation of this cell. Therefore, antioxidants ensure the protection and safety of the body by fighting the oxidation of cells in the body [34]. It is well known that oxidation can lead to many forms of cancer over time [35]. The existence in plants of compounds with antioxidant properties and high content of free radical scavenging compounds (carotenoids, polyphenolics, flavonics, anthocyanins, unsaturated fatty acids, vitamins, enzymes, and cofactors) has stimulated interest in their use in prophylactic and curative phytotherapy [36, 37].

Antioxidant activity of genistein was investigated with soybean asolectin encapsulated in a liposome (0-3.6 mg/mL of genistein) [38]. Genistein inhibited lipid peroxidation—thiobarbituric acid reactive substance (TBARS) method—induced by hydroxyl radicals in 90.5% in the used C6 rat glioma cell line. Several works using polymeric hemodialysis membranes such as polysulfone, polyethersulfone (PES), and polyvinylpyrrolidone (PVP) modified with genistein exhibit that these forms of encapsulation of hydrophobic isoflavone may be used to treat several diseases (neurodegenerative, cardiovascular, etc.). It was the measured generation of reactive oxygen species (ROS) levels by dihydroergotamine assay. Comparison with mangiferin-modified forms of genistein [39] demonstrated higher antioxidant properties at the doses 25 *μ*g/mL-200 *μ*g/mL. Mangiferin may show low activity due to the presence of the glucose unit that was exhibited in the test with genistin (genistein 7-*O*-glucoside), xanthone, and glucose test solution. The PES membranes with genistein exhibited better antioxidant properties related to polysulfone membranes with genistein (57% vs. 27%). In Chang et al.'s [40] report, it was demonstrated that modified membranes PES-PVP in the ratio 82.5 : 17.5 with genistein had higher antioxidant activity vs. unmodified PES-PVP (39% of the level of ROS for PES : PVP/genistein 90/10 vs. about 60% of the level of ROS for unmodified PES/PVP 90/10).

The effect of quercetin and genistein in the dose 10 or 20 *μ*M on human leukemia (U937) cells was investigated [41]. Oxidation was induced by iron or copper (50 *μ*M both) in H_2_O_2_ (0.01 mM). Also, the effect on the glutathione was measured with flavonoids (0-40 *μ*M). It was found that both treatments with quercetin and genistein for the Fe- or Cu-induced oxidative damage provide better protection to U937 cells. In the test of glutathione levels for quercetin, it was detected 4.5, 8.3, 11.7, and 15.02 nmol/10^6^ cells using 5, 10, 20, and 40 *μ*M; for genistein, it was 3.8, 7.9, 12.5, and 14.6 nmol/10^6^ cells (5, 10, 20, and 40 *μ*M). Quercetin was more active. Later, Boadi et al. [42] used mouse 3T3-L1 fibroblast cells and three flavonoids (quercetin, kaempferol, and genistein). Oxidation was induced using Fe (50 *μ*M) ions and H_2_O_2_ (0.01 mM). There were measured levels of reduced glutathione, glutathione peroxidase, glutathione reductase, and superoxide dismutase. In the results, glutathione levels decreased for quercetin at the 5, 10, and 25 *μ*M doses but did not change the same for the 15 and 20 *μ*M doses. For kaempferol and genistein, a similar effect was found. The levels of peroxide enzymes increased using all doses of flavonoids. The most active was quercetin.

*In vitro* antioxidant activity of genistein was observed in Huh7.5 (male immortalized human hepatocarcinoma cell line) and LX-2 (HSCs; male immortalized human hepatic stellate cell line) [43]. Also, antioxidant activity was observed *in vivo* in adult male Sprague-Dawley rats (40 mg/kg/day genistein) [44].

### 4.2. Angiogenesis

Tumor angiogenesis refers to the growth of new vessels around and inside tumors; this involves the proliferation and migration of endothelial cells so as to form new lumens, plexuses, and vascular networks [45, 46]. Genistein was investigated to induce angiogenesis at the concentration (0.001–100 *μ*M) in o [47]. In the low concentration (0.001–1 *μ*M), genistein induced angiogenesis by promoted tube formation. In contrast, at the high concentration (25–100 *μ*M), it inhibited pseudo-microvessel outgrowth. So, it is a double effect of genistein at the capillary formation.

Also, an *in vivo* study was carried out using the chorioallantoic membrane of the chicken embryo [48]. The decrease of angiogenesis was measured *in ovo* and *ex ovo* using a stereomicroscope. The concentration for each sample (genistein and three complexes of genistein and cyclodextrins, 1 : 1) was 10 mM. The most expressed effect was for genistein alone. In *in silico*, *in vitro*, and *in vivo* studies, the antiangiogenic genistein effect was corroborated [49, 50].

### 4.3. Anticancer and Cytotoxic Activity

**Figure 3:**
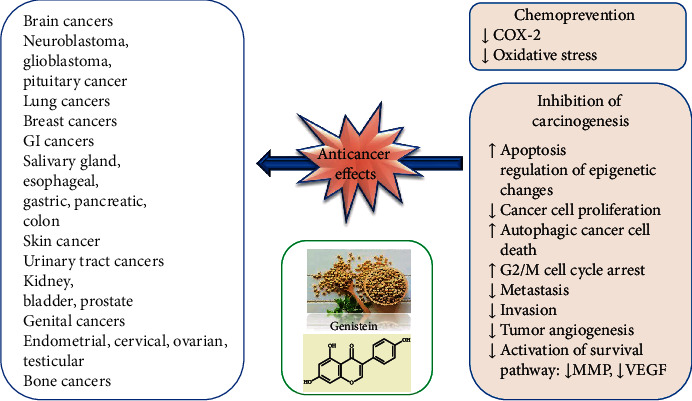
Summary of the main anticancer mechanisms of genistein.

Genistein has been proven preclinical effectual against various types of human cancers such as breast, lung, liver, prostate, pancreatic, skin, cervical, bone, uterine, colon, kidney, bladder, neuroblastoma, gastric, esophageal, pituitary, salivary gland, testicular, and ovarian cancers (Figure 3, Table 1).

#### 4.3.1. Brain Tumors

*(1) Neuroblastoma Cancer*. Neuroblastoma cancer generally occurs as an extracranial solid tumor [51]. Administration of the rapamycin (200 nM) induces autophagy in malignant neuroblastoma IMR-32 and SK-N-BE2 cells in humans [52]. The combining effect of microtubule-associated protein light chain 3 short hairpin RNA (LC3 shRNA) plasmid transfection (50 nM) and genistein (25 *μ*M) inhibited the rapamycin-induced autophagy, promoted the apoptosis, and decreased the cell viability. They also inhibited the autophagy-encouraging marker molecules (Myd88, Beclin 1, LC3 II, and TLR4) and upregulation of autophagy-reducing marker molecules (mTOR and p62) in both cell lines [52].

In human neuroblastoma SK-N-SH cells, the genistein (12.5 *μ*M) dose *in vitro* induces cell cycle arrest at phase G2/M and also eliminated the E2- or endocrine disruptor (environmental)-stimulated proliferation through the Akt pathway-dependent way [53]. Genistein is an epigenetic modifier that can inhibit hypermethylation levels and enhances the expression of CHD5, and p53 also contributes to the inhibition of neuroblastoma growth and tumor microvessel formation *in vivo*. Furthermore, genistein significantly inhibits the expression of DNMT3b and acts like a DNA methyltransferase (DNMT) inhibitor.

In the inhibition of neuroblastoma growth, genistein plays a vital role *in vivo* [54]. Genistein boosts the survival of neuroblastoma SK-N-SH cells to avoid 6-hydroxydopamine- (6-OHDA-) stimulated neurotoxicity in humans. At the G0/G1 phase, 6-OHDA causes cell arrest and prevented S-phase entry. Pretreatment of genistein on the cell cycle can reverse the cytostatic effect of 6-OHDA. The decrease in mitochondrial membrane potential stimulated by 6-OHDA can be reversed through pretreatment of genistein. Through cotreatment with JB-1, these effects can be blocked completely which is an antagonist of the IGF-1 receptor. Moreover, pretreatment of genistein restored the 6-OHDA-stimulated upregulation of Bax and inhibited Bcl-2 mRNA and protein expressions. Treatment of genistein alone can significantly induce ERE luciferase activity and boosts up phosphorylation levels of MEK. Combined treatment with IGF-1 can upregulate the genistein effect on MEK phosphorylation and cell proliferation [55]. Genistein treatment (10 nM to 10 *μ*M) for 20 min induced noradrenaline (NA) uptake through SK-N-SH cells. Genistein also induced uptake of serotonin and NA through the serotonin transporter and NAT transiently transfected COS-7 cells. The velocity of NA transport can also be significantly increased with no change or a little change in affinity. Maximal binding is also increased without changing the dissociation constant.

Genistein is also known as an inhibitor of daidzein, tyrosine kinases, and inactive genistein analogue that had some effects on NA uptake against tyrosine kinases by SK-N-SH cells. Through the treatment of tyrphostin 25, the stimulatory effects were observed on NAT activity. Tyrphostin 25 is an inhibitor of epidermal growth receptor tyrosine kinase, while the tyrosine phosphatase inhibitor (orthovanadate) restrained NA uptake by COS-7 cells (NAT transfected). It also upregulates neuronal monoamine transporter activity through protein tyrosine phosphorylation [56]. Bcl-2 siRNA and genistein combination caused more than an 80% decrease of cell proliferation in malignant neuroblastoma SK-N-DZ cells in humans. FACS analysis and TUNEL staining exhibited apoptosis in 70% of the cells after the combined treatment of both genes. Apoptosis was related to an increase in the mitochondrial release of cytochrome c, Bax : Bcl-2 ratio, and caspase activation through a mitochondria-mediated apoptotic pathway. Genistein activates the receptor-mediated apoptotic pathway by boosting up FasL, TNFR-1, tumor necrosis factor alpha (TNF-*α*), Fas-associated death domain (FADD), and also caspase-8 activation. The combined treatment of genistein and Bcl-2 siRNA triggered the increase in PARP and DFF45 cleavage that induces apoptosis [57].

There is synergistic efficiency of genistein and sorafenib (SF) combined treatment in human malignant neuroblastoma SH-SY5Y (N-Myc nonamplified) and SK-N-DZ (N-Myc amplified) cell lines. Combined treatment of Bid to tBid and caspase-8 increased the p21 and p53 expression, enhanced the Bax : Bcl-2 ratio, and downregulated the antiapoptotic Mcl-1 to trigger apoptosis. Downregulation of hTERT, VEGF, NF-*κ*B, c-IAP2, MDR, N-Myc, FGF2, and p-Akt showed suppression of survival and angiogenic pathways. In the cytosol, mitochondrial release of Smac and cytochrome c indicated mitochondrial involvement in apoptosis. In proteolytic activities, an increase of caspase-3 and calpain was also confirmed. The combination of genistein and SF inhibited survival and angiogenic factors and upregulated apoptosis through mitochondria- and receptor-mediated pathways in neuroblastoma SH-SY5Y and SK-N-DZ cell lines [58]. In human malignant neuroblastoma SH-SY5Y and SK-N-BE2 cancer cell lines, genistein and retinoid N-(4-hydroxyphenyl) retinamide (4-HPR) significantly inhibited tumor volume because of overwhelming apoptosis in neuroblastoma xenografts *in vivo*.

Combined treatment of genistein and 4-HPR can reduce tumor weight, body weight, and tumor volume in a time-dependent manner. Combination of genistein and 4-HPR boosts up the mitochondrial release of Smac and Bax : Bcl-2 ratio and inhibited the BIRC (baculovirus inhibitor of apoptosis repeat containing) proteins which also includes (BIRC-2 and BIRC-3) and stimulates AIF (apoptosis-inducing factor) and caspase-3. Moreover, inhibition of VEGF (vascular endothelial growth factor), FGF2 (fibroblast growth factor 2), and NF-*κ*B was also detected. Immunofluorescent labeling of the tumor section demonstrated overexpression of caspase-3, caspase-12, calpain, and AIF in apoptosis. The combined treatment enhances apoptosis in xenografts but did not stimulate liver and kidney toxicities in animals [59]. The combination of 10 *μ*M molecule Bcl-2-reduced HA14-1 (HA) and 250 *μ*M genistein in SK-N-BE2 cells was more efficient in stimulating apoptosis in cell lines (HA or genistein alone). The combined treatment of genistein and HA caused activation of Bax and inhibited Bcl-2 which increases the mitochondrial release of cytochrome c, AIF, Smac, and Bax : Bcl-2 ratio. Inhibition of survival factors like N-Myc, survivin, and NF-*κ*B promoted apoptosis. In the course of apoptosis, the activation of calpain, caspase-3, and caspase-8 occurred. Increased caspase-3 and calpain activities were proved in degradation of 120 kD SBDP and SBDP (alpha spectrin to 145 kD spectrin breakdown product) [60–62]. Genistein downregulated the growth of medulloblastoma and glioblastoma multiforme cells with different radioresponses and TP53 mutations by cell arrest at the G2/M phase in the cell cycle. This was not associated with DNA damage and proved that cell cycle arrest triggered did not cause apoptosis [63]. Genistein can reduce the activity of telomerase resulting in telomere shortening. Telomerase is the enzyme capable of maintaining the length of telomeres [64, 65]. Healthy cells produce telomerase in small amounts or not at all, so telomeres are progressively shortened until they reach a critical length, which triggers cell death or replicative senescence [66]. In brain tumor cells, genistein stimulates growth arrest in connection with telomerase inhibition through suppression of the RNA template and TERT mRNA [67].

*(2) Pituitary Cancer*. In Wistar rats (sixteen months old), genistein (30 mg per kg per day) directed subcutaneously modulated the immunohistomorphometric characteristics of ACTH cells for three weeks and reduced corticosterone levels and blood ACTH that supports the evidence that this isoflavone reduces glucocorticoid hormone secretion and also affects the hypothalamic-pituitary-adrenal axis [68].

In human prolactinoma cells, the dose of genistein (100 *μ*M) can enhance the percentage of cells in phase G1 from 55.3% to 90.3%. E2 of different concentrations can increase the proliferation of prolactinoma cells dose-dependently in humans. E2 (100 *μ*M) can enhance the percentage of cells in phase G2 from 15.6 to 41.8%. It inhibits DNA synthesis, cell proliferation, and the cell cycle of cultured pituitary cells in humans and induces apoptosis. On the suppression of proliferation, E2 partially inhibits the effect of genistein, not apoptotic stimulation of cultured prolactinoma cells *in vitro* [69]. Genistein inhibited the proliferation of mouse AtT-20 cells and rat anterior pituitary cells. Genistein (50 and 100 *μ*M) inhibited the AtT-20 cell proliferation at the G0/G1 phase and G2/M phase and induced an apoptotic peak of cells with 19.9% and 36.4% apoptotic ratios. Finally, genistein can significantly decrease the proliferation of cells (pituitary cells) as a tyrosine kinase inhibitor by stimulating apoptosis. And tyrosine kinase activity can play a vital role in the differentiation and proliferation of pituitary cells [70].

*(3) Breast Cancer*. In T47D and MCF-7-C3 breast cancer cells, genistein persuades downregulation of cancerous prohibitor of protein phosphatase 2A (CIP2A), which is associated with apoptotic activities and growth prevention in cells [71]. CIP2A overexpression was attenuated, whereas CIP2A knockdown sensitized the growth inhibition and apoptosis induced by genistein. It also stimulates downregulation of CIP2A concerned with both proteasomal degradation and transcriptional suppression. Specifically, at high concentrations, genistein stimulates circumstantial downregulation of CIP2A and E2F1 [71]. There is stimulation at the protein level of ATP-binding cassette subfamily C member 1 (ABCC1) and ABCG2 in MCF-7 and MDA-MB-231, respectively [72]. Depending on ABCG2 activity, MCF-7 cells demonstrate a parallel increase and resistance in mitoxantrone and doxorubicin efflux. Due to concurrent inhibition and ABCC1 induction by genistein, cells adapted neither chemoresistance nor drug efflux [72].

Morphological modification of mammospheres is promoted through the administration of genistein (2 *μ*M and 40 nM) in breast cancer stem cells (BCSCs) [73]. And it upregulates the appearance of cells of mammospheres in the coculture system and minimizes the ratio of the subset of CD44+/CD24-/ESA+ cells. From ER+ cancer cells, amphiregulin is released and that activates signaling pathways PI3K/Akt and MEK/ERK. On mammospheres, the differentiation-inducing effect is connected to these signaling pathways [73]. There have been different pieces of evidence that genistein represents anticancerous effects in triple-negative breast cancer (TNBC) by inducing G2/M cell cycle apoptosis and arrest [74]. On 226 proteins, genistein regulates phosphorylation sites. This data elaborates that throughout the cell cycles, genistein can control different biological processes including cohesion complex cleavage, DNA replication, and kinetochore formation.

Genistein can activate the BRCA1 and ATR complex and DNA damage response. In a more complex way, genistein is also able to slow down the TNBC growth of cells at the phosphoproteomic level by modifying the DNA damage and cell cycle [74]. Without disturbing the feasibility of nonmetastatic MCF-7 cells, genistein stimulates apoptosis in metastatic Hs578t and MDA-MB-435 cells and reduces cell viability in MDA-MB-435 cancer cells. Similarly, with reduced cell capability, miR-155 is downregulated while anticell proliferative miR-155 and proapoptotic PTEN, p27, FOXO3, and casein kinase, with genistein treatment, are upregulated in Hs578t and MDA-MB-435 cells. On the other hand, in MCF-7 cells, in response to genistein, miR-155 levels stay unaffected. In Hs578t and MDA-MB-435 cells, ectopic expression reduces the consequence of genistein on cell activity and abolishes the genistein effect on apoptosis and proapoptotic genes [75–77]. At the dose of 175 *μ*M, genistein upregulated the miR-23b in MCF-7 cells [78, 79].

In breast cancer cell development, cytochrome P450 1B1 (CYP1B1) plays a vital role by activating environmental carcinogens and endogenous estrogens [80]. At 5 *μ*M, a synergistic effect is produced through genistein on CYP1B1 mRNA levels stimulated by environmental carcinogen 7,12-dimethylbenz[a]anthracene (DMBA) [81]. From the second day of culture, the cellular level of ROS is increased and it also stimulated cell proliferation [81–83]. Genistein controls growth and stimulates cell apoptosis in MCF-7 cells. Moreover, genistein influences the inactivation of p-Akt and IGF-1R and also downregulated the B-cell lymphoma 2 (Bcl-2)/Bcl-2-associated X protein-protein ratio.

These results advocate that immobilizing the IGF-1R-PI3K/Akt pathway, genistein obstructs cell proliferation, lessening the protein expressions and Bcl-2/Bax mRNA [84, 85]. Likewise, genistein slows down proliferation and stimulates apoptosis in cells MCF-7 and T47D, particularly after calycosin treatment. MCF-7 cell treatment with genistein or calycosin causes a decrease in phosphorylation of Akt and reduces the expression of the downstream target, HOTAIR [84, 86].

In MCF-7/Adr cells, the combination of genistein with doxorubicin had a synergistic effect, and genistein reduced the chemoresistance of these cells [87]. Genistein has no special effect on P-gp function, but it boosts up the intracellular accumulation of doxorubicin. Doxorubicin and genistein combination considerably stimulated apoptosis and cell cycle arrest. Treatment of genistein reduces HER2/neu except for MDR-1 expression at both the protein and mRNA levels. Consequently, on MCF-7/Adr cells, doxorubicin and genistein combination had a collegial effect through inhibition of HER2/neu expression and doxorubicin increases in intracellular accumulation [87]. Through stimulation of apoptosis and ER-*α* expression regulation, various concentrations of genistein (50, 100, 150, and 200 *μ*M) for 24, 48, or 72 hours have an anticancer role in a concentration-dependent manner against 3T3-L1 and MCF-7 cells [88]. In a concentration-dependent manner, they considerably decreased. Through genistein, as Bax induced, the Bcl-2 expression was slowed down. The present study suggested by its results that the separation of 3T3-L1 cells and proliferation of MCF-7 induction of apoptosis and an ER-*α*-related pathway are involved [88]. Genistein exposure to MCF-7 cells stimulates the increase in intracellular levels of ribose 5-phosphate and 6-phosphogluconate, implying the upregulation of the pentose phosphate pathway. It causes a considerable decrease in glutamine and glucose uptake and strictly restricts their growth leading to variation in protein biosynthesis [89–94].

*(4) Lung Cancer*. Lung cancer is an increase in abnormal cells in the lung [95]. These cells multiply and grow at a faster rate than normal cells [96]. Different concentrations of genistein (0, 10, 25, 50, 100, and 200 *μ*M) were exposed to A549 cells for three consecutive days; they inhibit cell apoptosis in A549 cells and promote caspase-3/9 activation in a dose-dependent manner [97]. Further functional examination proved that genistein has an anti-cancer effect, and in A549 cells, it reduces MET protein expression and stimulated microRNA-27a (miR-27a) expression [97]. On NSCLC A549 cells, genistein particularly exhibits a radiosensitizing effect. Rather than MRC-5 cells, genistein induced oxidative stress in A549 cells, as determined by dichloro-dihydro-fluorescein diacetate (DCFH-DA) assay and oxidative damage marked by malondialdehyde (MDA), carbonyl protein, or 8-hydroxy-2′-deoxyguanosine (8-OHdG) content [98]. Genistein slows down the level of methylation and boosts up mRNA expression in the Keap1 promoter region in A549 instead of MRC-5 cells. Therefore, it effectively prohibits the simulation of Nrf2 to the nucleus which upregulates ROS and abolishes Nrf2-dependent antioxidants. In A549 cells, genistein upregulates the level of ROS particularly when united with radiation, while in MRC-5 cells, it downregulates the radiation-induced oxidative stress, probably by increasing the level of expression of glutathione, Nrf2, and heme oxygenase-1 (HO-1). Furthermore, in A549 cells, genistein increased significantly when combined with radiation but not in MRC-5 cells [98].

In A549, NCI-H460 (H460), and ABC-1 cells, genistein has an antitumor effect on TSA [99]. Genistein is associated with increased histone or nonhistone protein acetylation. In ABC-1 cells (p53 mutant), the accelerating effects of genistein were examined, but in H460 and A549 cells, it has decreasing effects. In A549 and H460 cells, genistein increased trichostatin A (TSA) and stimulated apoptosis as compared in ABC-1 cells. In H460 and A549 cells, the genistein effect was reduced after silencing p53 expression. Additionally, in H460 and A549 cells, genistein improved TSA which stimulates histone H3/H4 acetylation. And in H460 cells, genistein also boosts up p53 acetylation. On apoptosis and TSA-induced histone/p53 acetylation, the genistein enhancing effect is diminished by an inhibitor of anacardic acid and acetyltransferase. The expression of protein p300 is increased when genistein is combined with TSA in NCI-H460 and A549 cells. Moreover, it is also proved through many types of research that in A549 tumor-bearing mice, genistein has antitumor effects [99]. In human lung adenocarcinoma cells A549, genistein combined with all-trans retinoic acid (ATRA) has a slow-down effect on expressions of ICAM-1, Bcl-2, and MUC1 cells and applies the synergistic effect to slow down the invasion of A549 cells. It influences the expressions of various ongoing activities; for example, it affects the proteins related to apoptosis (Bcl-2 and Bax) and proteins related to the cell cycle (p-ERK1/2, Cdk4, and Rb), and also, in lung cancer cells A549, it downregulates the metastatic potential [100].

On small-cell lung cancer (SCLC) cell line H446, genistein has antitumorous effects through various methods such as migration ability, cell cycle arrest at the G2/M phase, stimulation of apoptosis, and downregulation of proliferation [101]. Furthermore, on H446 cells, genistein increases the antiproliferative effect of cisplatin. More importantly, genistein led to a decrease of FOXM1 protein and inhibited a series of FOXM1 genes that regulate apoptosis including cyclin B1, cdc25B, survivin, and various cell cycles. Before genistein treatment through cDNA transfection, an increase in FOXM1 can downregulate H446 proliferation inhibition. Hence, for the very first time, the genistein effect is demonstrated in this study to have numerous antitumor effects in the H446 cell line arbitrated by the inhibition of FOXM1 [101]. In the same way, the feasibility of A549 cells is reduced by the 7-difluoromethyl-5,4′-dimethoxygenistein (dFMGEN) derivative of genistein in a concentration- and time-dependent manner and stimulates apoptosis at the G1 phase [102]. Cell cycle arrest of the G1 phase was associated with a considerable reduction of cyclin D1 and Cdk4 protein levels. Inhibition of cyclin D1 and cyclin-dependent kinase (Cdk)4 protein levels was the result of the increase of p15, p21, and p27 levels, and Rb protein phosphorylation was directly suppressed, and then the progression of the cell cycle was arrested [102].

In lung cancer cells A549, it also increases apoptosis stimulated by TSA [103]. By increasing the death receptor signaling of TNF receptor-1 (TNFR-1), the mechanism of apoptosis can be upregulated. At 5 and 10 *μ*M levels, genistein can significantly reduce the cell number and cause cell arrest in a dose-dependent manner when stimulated with TSA. Protein and mRNA expressions of TNFR-1 can be upregulated after combined treatment of TSA with genistein at 12 and 6 hours, respectively, when results were compared with the control group where TSA alone was used. About a 70% to 40% increase in TNFR-1 mRNA and protein expressions was witnessed when 10 *μ*M of genistein combined with TSA was used, respectively. Activation of p53 protein and caspase-3 and caspase-10 was also upregulated after combined treatment in A549 cells. In A549 cells, the expression of caspase-3 was downregulated by inhibiting TNFR-1 expression and a decrease in the cell number was the result of genistein and TSA [103].

#### 4.3.2. Gastrointestinal Cancers

*(1) Salivary Gland Cancer*. Salivary gland cancer is malignant (metastatic) and is the excessive growth of salivary gland cells [104]. This type of cancer is part of the so-called head and neck cancer (this group of cancers includes cancer of the oral cavity, salivary glands, paranasal sinuses, nasal cavity, pharynx, larynx, lymph nodes, and salivary adenoid cystic carcinoma- (SACC-) 83 cell line); an increase in genistein concentration (220 *μ*M) for 3 days can significantly increase the Bax protein expression and decreases the expression of survivin and Bcl-2 proteins [105]. In the same way, genistein inhibited proliferation in the SACC-83 cell line and the protein tyrosine kinase inhibitor shows antiproliferation effects. Treatment of genistein (220 *μ*M) for 72 hours reduces the growth of the SACC-83 cell line in humans; it stimulates apoptosis and decreases the survivin expression [106]. Furthermore, genistein also inhibits the cyclin D1, cyclin B1, Cdk4, and Cdk1 protein expression in SACC-83 cells. Treatment with genistein (220 *μ*M) for 72 hours induces a decrease in the expression level of Cdk4, cyclin D1, cyclin B1, and Cdk1 which was 43%, 46%, 58%, and 64%, respectively. In the SACC-83 cell line of humans, genistein induces G2/M cell cycle arrest that may be correlated with inhibition of the cyclin D1, cyclin B1, Cdk4, and Cdk1 protein expression [107]. It also stimulated cell apoptosis that leads to a conclusion that protein tyrosine kinase induces an important effect on the growth of SACC and on neoplasia [108]. Genistein has a significant effect on SACC *in vivo*, though it exhibited an inhibitory effect on metastasis. In nude mice, genistein stimulated apoptosis, which decreases the expression of MMP-9 and VEGF on SACC [109].

*(2) Gastric Cancer*. Gastric cancer is formed from a cell that is part of the structure of the stomach. Most gastric cancers originate in the cells that make up the gastric mucosa (the cell lining that lines the inner surface of the stomach) [110]

In esophageal squamous cell cancer, TE-2 (p53, wild) and TE-1 (p53, mutant) cell lines in human genistein (30 *μ*M) upregulate the radiosensitivity of cell lines by suppressing the p42/p44 extracellular signal of regulated kinase and radiation-stimulated activation of the survival signal and Akt/PKB [111, 112]. In TE-2, a significant increase in the poly(ADP-ribose) polymerase cleavage and percentage of apoptotic cells was observed, but in TE-1, no change was seen even after the combined treatment of irradiation and genistein. In p53-related proteins, the expression of Bax was increased, but in Bcl-2, a decrease in expression was observed clearly in TE-2 but the opposite in the TE-1 case. This suggests that the main approach of cell death in a cell line was stimulated through genistein with wild-type p53 which varied from mutant p53 [111, 112].

Genistein with 15 *μ*M concentration decreases chemoresistance to 5-FU and cisplatin [113]. Other results also proved that reduced chemoresistance can be related to inhibition of ERK1/2 activity and ABCG2 expression. Moreover, in the xenograft model, genistein also reduces the tumor mass. Together, genistein decreased the cell-like properties of gastric cancer stem cells and inhibited chemoresistance [113]. Genistein can transform typical cellular characteristics in stem cells of cancer by inhibiting signaling Gli1-related pathways. CD44(+) cells demonstrated cancer stem-like cell properties and created sphere colonies. Additionally, in CD44(+) cells, sonic hedgehog (Shh) signaling genes were upregulated when compared with CD44(-) cell levels. When cancer stem-like cells (CD44(+)) treated with genistein inhibited CD44 mRNA and Gli1 protein expressions. Furthermore, genistein also inhibited stem cell markers; Gli1 siRNA confirmed the genistein action in reducing Gli1 expression. The high cell CD44(+) migration capacity was inhibited by genistein. It reduces Gli1 gene expression, and in gastric cancer cells, it decreases cancer stem-like properties. The cell invasive ability was suppressed through genistein that is required for metastasis and tumor growth [114]. On cell proliferation, genistein shows inhibitory effects that are associated with inhibition of cdc2 activities and G2/M cell cycle arrest. In gastric cancer (BGC-823 and SGC-7901) cell lines, genistein stimulated dose-dependent accumulation in the G2/M phase of the cell cycle. In BGC-823 and SGC-7901 cells, genistein sustained G2/M arrest which is related to inhibited cdc2 protein and increased phospho-cdc2 (Tyr15).

Treatment of genistein inhibited phospho-Wee1 (Ser642) and upregulated the Wee1 levels. Genistein substantially upregulated PTEN expression and inhibited Thr308 and Ser473 phosphorylation of Akt. Combined treatment of genistein with siRNA downregulated PTEN, phospho-cdc2 (Tyr15), and G2/M cell cycle arrest, therefore increasing phospho-Wee1 (Ser642). Genistein stimulation of G2/M cell arrest involved an increase in PTEN [115].

Genistein shows its anticarcinogenic effects through apoptosis and by inducing G2/M arrest of cancer cells. In gastric cancer cells, it stimulated protein alterations and changed the molecular mechanism which is responsible for actions (anticancerous) of genistein. Through genistein, a total of 86 proteins were regulated, most of which were combined into G2/M transition and regulation of cell division, consistent with effects (anticancer) of genistein. Various proteins CDCA8, CIT, and TPX2 (including kinesin family proteins) were regulated by genistein. Five kinesin family proteins CENPF, KIF23, KIF22, KIF20A, and KIF11 were significantly downregulated by genistein. Considerably, decreased KIF20A was chosen for further functional studies. The silencing of KIF20A reduces cell viability and stimulated G2/M arrest just like the effects of genistein in gastric cancer. The silencing of KIF20A also enhances the sensitivity of cancer cells to genistein inhibition whereas KIF20A overexpression markedly reduces genistein-stimulated cell viability and G2/M arrest [116].

Genistein is mediated through the suppression of COX-2 and explained the mechanism of action in BGC-823 cells. Treatment with genistein induced apoptosis and inhibited cell proliferation in a time- and dose-dependent manner. Genistein treatment applied a significant inhibitory effect on NF-*κ*B activation. In addition, the NF-*κ*B downregulated pyrrolidine dithiocarbamate leading to a reduction of COX-2 protein levels and activation of NF-*κ*B-like genistein effects. COX-2 protein suppression may be important for proapoptotic and antiproliferative effects in BGC-823 cells, and through the NF-*κ*B pathway, these effects may be moderately changed [117].

In AGS, 5Aza-C and genistein stimulated PCDH17 mRNA expression but not in Ges-1. Furthermore, in AGS, the combined treatment of 5Aza-C and genistein can significantly inhibit promoter methylation and reactivated PCDH17 expression in putative methylation target regions [118, 119].

In human gastric cancer AGS and SGC-7901 cell lines, genistein analogue 7-difluoromethoxyl-5,4′-di-n-octylgenistein (DFOG) inhibited colony formation and cell viability of AGS and SGC-7901 cells. Moreover, in the G2/M phase, DFOG significantly arrested the cell cycles. DFOG reduces the FOXM1 expression and its downstream genes (cdc25B, cyclin B, and Cdk1) and boosts up p27Kip1 at protein levels. By small-interfering RNA, knockdown of FOXM1 resulted in increased cell growth inhibition in various AGS cells before DFOG treatment. By cDNA transfection, upregulation of FOXM1 attenuated DFOG-stimulated cell growth inhibition in various AGS cells [120].

The combination of genistein and TRAIL stimulated sub-G1 phase DNA content and chromatin condensation. These apoptosis markers are related to death receptor (DR5) activation and stimulation of caspase-3 activity that results in cleavage of poly(ADP-ribose) polymerase. Both apoptotic characteristics and cytotoxic effects stimulated by cotreatment were significantly reduced by a caspase-3 inhibitor, Z-DEVD-FMK, which explains the role of caspase-3 in cytotoxic effects [121].

*(3) Liver Cancer*. Genistein controls the metastasis process, the main cause of liver cancer. This process may be controlled by levels of epithelial factors *α*-catenin, E-cadherin, and mesenchymal factors N-cadherin and vimentin. Genistein decreases levels of mesenchymal factors and increases levels of epithelial factors. Also, it inhibits the process of transforming growth factor-beta- (TGF-*β*-) induced epithelial-mesenchymal transition (EMT), the main pathway of the distribution of metastasis. Also, genistein may induce apoptosis of cells by adhesion molecules such as focal adhesion kinase (FAK) which plays a major role in the integrin-mediated signal transduction pathway [122].

In rats, fulminant hepatic failure (FHF) is instigated through D-galactosamine (D-GalN) 250 mg/kg body weight (BW) when it is used twice a week for 12 weeks. The effect of genistein (5 mg/kg BW) significantly attenuated D-GalN-induced chronic damage and fibrosis in the liver as evident from a significant amelioration in functional impairment, including inhibition of the activation of HSC, decreased expression in alpha-smooth muscle actin (*α*-SMA) and accumulation of the collagen matrix, and increased serum alanine transaminase (ALT) and aspartate transaminase (AST) levels [123, 124]. Furthermore, combined treatment of genistein with hepatic Smad7 expression downregulates the TGF-*β* expression and stimulates TGF-*β*/Smad signaling. Genistein also prevented significant histopathological modifications stimulated by D-GalN [123, 124]. Different doses of genistein 1, 10, 25, 50, 75, and 100 *μ*M can slow down cancerous cell growth in the hepatocellular carcinoma PLC/PRF5 cell line when used in particular times 24, 48, and 72 hours in a dose- and time-dependent manner [125]. In genistein treatment, the percentage of living cells was 47%, 48%, and 53% when used with 25 *μ*M concentration at various times. And at this concentration (25 *μ*M), genistein stimulates apoptosis or cell arrest particularly in a time-dependent manner. At different times, the percentage of the apoptotic cells was 44%, 56%, and 60%, respectively [125]. Genistein at different concentrations of 1.0 and 10 *μ*M in hepatocellular carcinoma (HCC) induces MRP2 mRNA and P-gp protein expressions and increases its activity. Genistein stimulated MRP2 mRNA and P-gp protein expressions at the concentration of 10 *μ*M, but at 1.0 *μ*M concentration, it does not induce any effect depending on the concentration-dependent manner [125]. Due to translational regulation of MRP2, inhibition of miR-379 expression by genistein can be observed. Through genistein, the silencing of pregnane X receptor (PXR) and stimulation of abolished P-gp (at 1.0 and 10 *μ*M) and MRP2 (only at 10 *μ*M) are also observed [126]. Through PXR and ERs, genistein puts its genomic effect and regulates different transporters. With better resistance to sorafenib cytotoxicity, genistein at 1.0 and 10 *μ*M concentrations can increase MRP2 and P-gp activity and expression. Stimulation of both transporters by genistein with 1.0 *μ*M concentration was downregulated through cycloheximide signifying translational regulation. Genistein inhibition of miR-379 expression can be connected with translational regulation of MRP2. Suggesting limited arbitration of genistein through PXR, when it is used at 1.0 and 10 *μ*M concentrations, it may cause the silencing of pregnane X receptor by GNT stimulation of abolished P-gp and MRP2 [126]. In HepG2/C3A cells, genistein can cause both long-term (72 h) stimulation and short-term downregulation of CYP1A activity. In male hormone cells, various enzyme activities and CYP1A gene expressions were encouraged to a greater extent rather than in female hormone cells [127, 128].

In human murine embryonic liver cells (BNL CL2) and hepatoma cells (Huh7, HepG2, and HA22T), genistein reduces transcription of matrix metallopeptidase- (MMP-) 9 by downregulating NF-*κ*B activity and activator protein- (AP-) 1 [129]. It silenced 12-*O*-tetradecanoylphorbol-13-acetate- (TPA-) stimulated AP-1 activity by reducing c-Jun N-terminal kinase (JNK) and extracellular signal-related kinase (ERK) pathways, and TPA induced inhibition of NF-*κ*B from inhibitory signaling pathways (I*κ*B). Furthermore, it also inhibits the TPA-stimulated activation of phosphatidylinositol/ERK3-kinase/Akt and boosts up the stream of activator protein and nuclear translocation [129]. In a dose-dependent manner, genistein increases *α*-catenin and E-cadherin in hepatocellular carcinoma SMMC-7721, HepG2, and Bel-7402 cells and decreases vimentin and N-cadherin at protein and mRNA levels. At the same time, genistein treatment downregulates EMT stimulated through TGF-*β*.

Genistein decreases protein and mRNA expressions in HepG2 cells and activated T cells (NFAT1), autotaxin, cyclooxygenase- (COX-) 2, ABCA3, and CD154; it reduces the nuclear factor. Ionomycin and phorbol 12-myristate 13-acetate (PMA) improve the activity of NFAT1 and inhibit *α*-catenin and E-cadherin protein levels and enhance vimentin and N-cadherin protein levels [130]. On HepG2 cells, downregulation effects of genistein demonstrated by transwell that ionomycin and PMA inverted the migration. Genistein reduces the intrahepatic metastasis by reversing EMT which was connected with reduced NFAT1 *in vivo*. Genistein mediated by NFAT1 restrained hepatocellular carcinoma cell migration by reversing the EMT [130]. Proliferation and growth of HCCLM3 cells are inhibited by genistein through eradicated cisplatin-induced MMP-2 expression. On HCC cell regularity and proliferation, genistein emphasized the lesser effect of cisplatin in nude mice after curative hepatectomy, perhaps through the improvement of cisplatin-stimulated MMP-2 upregulation [131, 132].

*(4) Pancreatic Cancer*. Through increased apoptosis, genistein boosts up 5-fluorouracil- (5-FU-) stimulated cell death as well as autophagy [133]. Due to inhibited Bcl-2 and improved Beclin 1 protein levels, autophagy was decreased. Other different studies like animal treatment also supported these observations. The combination of genistein and 5-FU considerably inhibited the final xenograft tumor volume compared to 5-FU alone by stimulating autophagy as well as apoptosis [133]. The expression of miR-27a is significantly inhibited through genistein in pancreatic cells. Furthermore, in pancreatic cells, inhibition of miR-27a reduces cell growth and invasion as well as induced apoptosis. Moreover, the combined effect of genistein and miR-27a on cell growth inhibition, invasion, and apoptosis implied that targeting miR-27a may be a potential strategy for pancreatic cancer treatment [134].

Genistein can inhibit onco-miR-223 which reduces cell growth and invasion and stimulation of apoptosis in pancreatic cancerous cells. The miR-223 expression is significantly inhibited by genistein treatment and Fbw7 induction that is one of the targets of miR-223. Likewise, the inhibition of miR-223 reduces the growth of cells and stimulated apoptosis in pancreatic cancerous cells [135]. Genistein upregulates miR-34a which led to inhibition of Notch-1 that is related to apoptosis induction and cell growth reduction in pancreatic cancerous cell lines. The proliferation of pancreatic cancer cells suppressed by miRNA could act like a nontoxic activator [136]. By regulating the protein expression of MMP-2 and uPA and mRNA, genistein can downregulate TGF-*β*1-stimulated metastasis and invasion in Panc-1. Simultaneously, genistein can also improve EMT progress through upregulation of vimentin and inhibition of E-cadherin [137].

*(5) Colon Cancer*. Colon cancer develops in the cells that line the colon and occurs when cells at this level, which are usually healthy, begin to grow uncontrollably, forming tumors [138]

In a DMH- (1,2-dimethylhydrazine-) stimulated group of rats, treatment of genistein inhibited the analytical indicator argyrophilic nucleolar organizer region (NOR) and PCNA (proliferating cell nuclear antigen) [139]. DMH administration stimulated oxidative stress, while genistein induces Nrf2 and inhibited target HO-1. Colonic stem cell indicator CD44, *β*-catenin, and CD133 protein expressions were upregulated in a DMH-stimulated group of animals when compared to the control group in rats [139]. In humans, genistein inhibited MMP-2 and Fms-related tyrosine kinase 4 (FLT4; vascular endothelial growth factor receptor 3) in CRC (colorectal cancer) tumors of mice. After indicating that genistein downregulated neoangiogenesis in the mouse tumor, it was also examined that in primary CRC, a significant increase in FLT4 expression was related to the decreased survival and increased stage [140]. In human colon cancer HT-29 and LoVo cells, genistein could stimulate apoptosis by downregulating the NF-*κ*B pathway and Bcl-2 while upregulating Bax; therefore, it provides the basis for genistein clinical application in colon cancer cases [141]. Genistein treatment (0-100 *μ*M) reduces cell proliferation, stimulates apoptosis and G2/M cell cycle arrest in the colon cancer HCT116 cell line, with a decline in mitochondrial membrane potential, and enhances intracellular ROS levels [142, 143]. Daidzein (50-100 *μ*M) and genistein (25 *μ*M) treatment inhibited the proliferation of the HT-29 cell line in grade II human colon adenocarcinoma. The concentration of genistein (50 *μ*M) suppressed *β*-catenin (CTNNBIP1) concentration [144]. Genistein transforms cell cycle distribution by accretion of cells at the G2/M phase with the considerable decreasing effect of serine/threonine-protein kinase 2 (Chk2) and cyclin B1 protein expressions in human Caco-2 (intestinal colon cancer) cells. In human colon cancer (SW480 and HCT116) cells, daidzein, biochanin A, and genistein showed growth inhibitory effects and promoted apoptosis.

However, genistein exhibited a significantly greater effect when compared with daidzein and biochanin A in a dose- and time-dependent manner. Additionally, in the G2/M phase, the genistein effect causes cell cycle arrest, the activation of p21waf1/cip1, GADD45*α*, and ATM/p53, and the inhibition of cdc2 and cdc25A. Genistein also stimulated cell cycle arrest of G2/M in a p53-dependent manner [145]. Genistein and soy protein isolate (SPI) have epigenetic effects on genes restraining their gene expressions stimulated by azoxymethane (AOM). In the post-AOM period, histone H3 acetylation (H3Ac) was inhibited through genistein and SPI at the promoter region of different genes (Sfrp2, Sfrp5, and Wnt5a) which are similar to the decreased binding of RNA polymerase II. The nuclear level of H3Ac was upregulated through genistein and SPI.

Diets inhibited phosphorylation of the histone H3S10P and trimethylation of the histone H3K9Me3. Methylation of the particular region of genes (Sfrp2, Sfrp5, and Wnt5a) was increased by genistein and SPI, which was inversely correlated with inhibition of gene expression [146].

Treatment of genistein in the human colon cancer SW480 cell line persuaded concentration-dependent G2-phase detention and inhibited cell proliferation. Overexpression of DKK1 established its contribution in growth inhibition, and inhibition of DKK1 expression slightly induced cell growth by siRNA. DKK1 gene expression was upregulated through genistein in HCT15 and SW480 cells. At the DKK1 promoter, DNA methylation was not affected by the treatment of genistein in all cell lines tested. In HCT15 and SW480 cells, histone H3 acetylation of the DKK1 promoter region was induced by genistein. Upregulation of histone acetylation is related to genistein-stimulated DKK1 expression. The alliance between DKK1 gene expression and histone acetylation is established by histone deacetylase inhibitor treatment TSA [147]. Injection of AOM stimulated aberrant nuclear accretion of colon cancer (*β*-catenin) cell lines of rats. Genistein inhibited Wnt target genes (c-Myc and cyclin D1) and also suppressed the expression of Wnt signaling genes including Sfrp1, Sfrp2, Sfrp5, and Wnt5a. It also reduces the number of the total aberrant crypts. Inhibition of Wnt/*β*-catenin signaling is related to a decrease in the entire aberrant crypts. The main role of genistein is an inhibitor of cancerous stimulated Wnt/*β*-catenin signaling in reducing the growth of early colon neoplasia [148, 149]. In colon cancer cells, genistein reduces epidermal growth factor- (EGF-) stimulated proliferation, though favoring nuclear maintenance of FOXO3 (active state) and dephosphorylation [148, 150, 151].

#### 4.3.3. Urinary Tract Cancers

*(1) Kidney Cancer*. The involvement in renal tissue injury in carcinogenesis in a chronic context has been proven. The process of tissue regeneration induced cellular lesions and involves mitosis and polyploidy, with altered cell function and the possibility of developing cancer cells [152]. In renal tubular cells (epithelial HK-2) of humans, parathyroid hormone (0.1 nM) treatment on cells for 48 hours can induce significant *α*-SMA protein expression and downregulated the protein expression of E-cadherin [153]. That treatment also increased protein expression and promoter activity of CTGF (connective tissue growth factor gene) and its mRNA. Significantly, in a dose-dependent treatment, genistein inhibited PTH-stimulated *α*-SMA expression, reduced CTGF protein and mRNA expressions, restored E-cadherin expression, and suppressed the activity of CTGF. Renal tubular epithelial cells of human genistein can block the biomarker for epithelial-mesenchymal alteration and renal transdifferentiation, *α*-SMA, following the treatment of PTH and inhibition of CTGF expression [153]. While inhibiting renal cell carcinoma (RCC) cell invasion and proliferation, genistein boosts up apoptosis and downregulated activity of TCF in RCC cells. Genistein highly expressed and significantly inhibited the miR-1260b in RCC cells [154]. In renal cancer tissues, the miR-1260b expression was significantly higher compared with normal tissues and was significantly associated with overall shorter survival. Additionally, in RCC cells, miR-1260b encouraged renal cancer cell invasion and proliferation [154]. In renal cancer cells (miR-1260b inhibitor transfected), the 3′UTR luciferase activity of DKK2, Sfrp1, and Smad4 target genes was significantly decreased and upregulated the protein expression. In some malignancies, BTG3/ANA/APRO4 was considered to be a gene that suppresses tumors. The combination of 5-Aza-2′-deoxycytidine (5Aza-C) and genistein stimulated the expression of BTG3 mRNA (messenger RNA) in A498, HEK-293, and ACHN in RCC cell lines. The treatment of genistein and 5Aza-C significantly inhibited promoter methylation that leads to activation of BTG3 expression. Genistein and 5Aza-C boost up the level of 2H3K4, acetylated histones 3 and 4, RNA polymerase II, and 3H3K4 at the BTG3 enhancer indicative of dynamic histone modification. The treatment of genistein and 5Aza-C inhibited the activity of methyl-CpG binding domain 2 and DNA methyltransferase and boosts up the activity of HAT [155, 156]. Genistein treatment in BRCA1 mutant cells decreased the G1 cell population that was accompanied at G2 by cell accumulation. Some cells that are treated with genistein entered mitosis, though they revealed chromosome abnormalities and sustained tetraploidy due to the abortive mitotic exit. The fraction of G2 cells undergo endoreduplication and turn out to be polyploidy, which was accompanied by apoptosis and activation of DNA damage response [157]. In RCC cells, genistein (100 *μ*g/mL) inhibited cell proliferation for 48 hours in a dose- and time-dependent manner. Genistein used with a dose of 50 *μ*g/mL significantly induced cell apoptosis. After establishing the Millipore filter chamber, vascular volume in RCC cells boosts up to threefold than without renal cell carcinoma cells. Genistein significantly decreased neovascularization in the Millipore filter chamber stimulated by human RCC cells [158].

*(2) Bladder Cancer*. Genistein in a dose-dependent manner could significantly sensitize various BDEC cells and bladder cancer cell lines to hydroxycamptothecin- (HCPT-) stimulated apoptosis both *in vivo* and *in vitro* [159–161]. Genistein and HCPT significantly inhibited proliferation and bladder cell growth and stimulated cell cycle arrest of the G2/M phase and apoptosis in BDEC cells and TCCSUP bladder cancer cells. Significantly, in the bladder cancer xenograft model, genistein attenuated HCPT ability to stimulate the NF-*κ*B pathway and activates antiapoptosis both *in vivo* and *in vitro*. Furthermore, the NF-*κ*B pathway also neutralized the antiapoptotic effect [159–161]. In human bladder cancer cell 253J B-V, genistein reduces the growth of cells in a dose- and time-dependent manner through G2/M arrest, inhibits NF-*κ*B, and induces apoptosis. In research, when mice were treated with genistein, it decreases final tumor weights which were related to stimulation of tumor cell apoptosis and reduces tumor angiogenesis *in vivo* [162, 163].

*(3) Prostate Cancer*. Against prostate cancer, genistein has shielding effects; using three prostate cancer cell lines LAPC-4, LNCaP, and PC-3 changes ER-*β* expression on the ER-*β* promoter methylation process [164–166]. In LNCaP and LAPC-4 cells, genistein (0.5-10 *μ*M) abridged ER-*β* promoter methylation depending on the dose, but in PC-3 cells, due to low basal levels of ER-*β* promoter methylation, the case is different from the other two cells. In all three prostrate cancerous (PCa) cells, genistein persuaded nuclear translocation, phosphorylation, and transcriptional activity of ER-*β*. Using specific ER-*β* antagonists on PC-3 and LAPC-4 cells, the production inhibitory effects of genistein were reduced. Genistein and ER-*β* work together to inhibit PCa cell proliferation by reducing promoter methylation; genistein boosts up the level of ER-*β*; meanwhile, ER-*β* mediates protective actions of genistein [164–166]. Genistein inhibits the miRNA-1260b expression in PCa cell lines [167]. In prostate cancer cells, while inhibiting the proliferation and transfection grade of T cell factor (TCF) reporter activity, genistein supports apoptosis. In prostate cancer cell tissues, miR-1260b was highly expressed and inhibited by genistein. In prostate cancer cells, after downregulating miR-1260b invasion, cell proliferation, TCF reporter activity, and migration were decreased. Western analysis and 3′UTR luciferase evaluated that miR-1260b was directly responsible for the regulation of two target genes (Smad4 and Sfrp1). In the prostate, cancerous tissue expression of Smad4 and Sfrp1 genes was particularly decreased. Through histone modifications and DNA demethylation, genistein enhanced Smad4 and Sfrp1 gene expression. In prostate cells, by inhibiting miR-1260b, genistein induces antitumor effects that targeted Smad4 and sRRP1 genes. Genistein also balanced Smad4 and Sfrp1 gene expression in prostate cancer cell lines through histone modification and DNA methylation [167]. In a dose-dependent manner, genistein reduces androgen receptor (AR) nuclear localization, LAPC-4 expression that has wild AR, and cell proliferation. When genistein is used with 0.5-5 *μ*M concentrations, it induces the growth of cells and boosts up AR expression and transcriptional activity. Inhibitory effects of genistein with higher doses were also examined by many researchers. In PC-3 cells, parallel results were attained and these cells were transfected with W741C, T877A, and H874Y AR mutants [168].

Genistein activates apoptotic signals and increases proapoptotic Bax protein expression, and in mCRPC cells, it boosts up the cabazitaxel treatment response. Combined treatment of cabazitaxel and genistein in the PC-3-luciferase xenograft model radically downregulated the mCRPC growth in comparison to genistein, vehicle control, or cabazitaxel [169]. lncRNA profiling demonstrated that genistein highly regulated HOTAIR and castration-resistant PCa cell line expression was higher than usual PCa cells. Cell proliferation, cell cycle arrest, apoptosis, and migration of prostate cancer cells were decreased due to inhibition of HOTAIR. In both DU145 and PC-3 prostate cancer cells, HOTAIR was directly targeted by genistein and it also upregulated miR-34a expression. Prostate cancer cell growth was also affected by inhibiting oncogenic HOTAIR which is influenced by tumor suppressor miR-34a [170].

Different concentrations of daidzein were used on cells, genistein (25-200 *μ*M) alone or with various combinations (25 or 50 *μ*M), and apoptosis, cellular uptake of isoflavones, cell proliferation, and cell cycles were measured after 48 hours [171]. Genistein and daidzein demonstrated a synergistic effect on restraining cell proliferation and stimulating apoptosis in PCa (C4-2B and LNCaP) cells [171]. Genistein reduces cancerous cell growth by gene modulation controlling cell cycle development. Genistein inhibits the kappa light polypeptide gene activation and enhances B-cells (NF-*κ*B), a signaling pathway that is implicated in the balance between cell survival and apoptosis [172]. Genistein (25 *μ*M) treated DU145 and PC-3 cells and inhibited miR-151 expression compared with the vehicle control. In prostate cancer cells, inhibition of miR-151 expression significantly downregulated invasion and cell migration [173]. Various genes IL1RAPL1, N4BP1, ARHGDIA, SOX17, and CASZ1 have tumor-suppressive roles which were target genes of miR-151 [173, 174]. Colony formation and tumorsphere formation of prostate cancer cells suppressed through genistein *in vivo*. It accelerates the inhibition of prostate cancer cell marker CD44 *in vivo* and *in vitro* and also downregulates the hedgehog-Gli1 pathway that contributes to the anticancer stem cell effect of genistein in prostate cancer TCs [175]. The combining effects of topotecan and genistein in prostate cancer cells (LNCaP) can stimulate cell death, inhibiting cell viability (LNCaP), and apoptosis through activation of caspase-3 and caspase-9 that are engaged in intrinsic pathways. With topotecan and genistein combined treatment, levels of ROS generation significantly increased [176, 177].

#### 4.3.4. Genital Cancers

*(1) Uterine Endometrial Cancer*. In endometrial epithelial cells, genistein upregulated Toll-like receptor 2 (TLR2) and reduced the viral component-stimulated TLR2 protein expression [178]. In three cell (uterine sarcoma) lines, genistein with 9.3 *μ*M, 13.1 *μ*M, and 19.2 *μ*M concentrations has inhibitory effects on MES-SA, MES-SA-Dx5, and SK-UT-1, respectively through stimulation of DNA fragmentation and Dickkopf-related protein 1 (DKK1) and induction of p53. Also, in all three lines, we have suppression of Dishevelled protein (DVL), survivin, histone deacetylase 4/5/7 (HDAC4/5/7), Bax, and phosphorylated mitogen-stimulated protein kinase kinase (phospho-MEK); inhibition of *β*-catenin and p27 in more resistant lines SK-UT-1 and MES-SA-Dx5; inhibition of ERKs and protein kinase B (Akt); and phosphorylation and stimulation of caspase-3 in MES-SA-Dx5 and MES-SA parental derived lines. Inhibition of *β*-catenin expression also corresponded with decreased activity in TOPFlash [179, 180]. In endometrial cancer (Ishikawa) cells, the combination of genistein and indole-3-carbinol (I3C) demonstrated a significant increase in cell death and sub-G1 arrest. A combination of three treatments proved eminent expression of DR4, DR5, and cleaved forms of caspase-8, caspase-3, and PARP; the flip was inhibited. Furthermore, upregulation of DNA fragmentation and caspase-3 activity indicated the stimulation of apoptosis. Genistein and I3C with TRAIL synergistically stimulated apoptosis through the death receptor-dependent pathway [181].

Estrogen replacement therapy is connected with an increased risk of breast cancer and uterine cancer. In the H19-7/IGF-1R neural cell line, genistein, daidzein, and E2 ranging from 20 to 2000 nM considerably promoted proliferation and hippocampus neuronal cell viability. In the S phase, daidzein and genistein stimulated an increased effect on the percentage of cells just like E2. Daidzein and genistein significantly boost up the protein level and BDNF mRNA expression [182]. In cell proliferation of uterine leiomyoma (UtLM), the concentration of genistein 50 *μ*g/mL inhibited members of the TGF-*β* pathway and downregulated expression of protein activin A and Smad3 [183]. Administration of genistein (0.5 mg/kg body weight subcutaneously) downregulated E2-stimulated mitoses in the endometrial stroma, uterine luminal epithelium, and myometrium and also partially inhibited endometrial edema and E2-stimulated uterine eosinophilia. In the uterus, it provides defense against estrogen-stimulated cell proliferation [184].

In endometrial adenocarcinoma (Ishikawa) cells, both genistein and estrogen upregulated ER-stimulated gene activity and decreased estrogen-induced proliferation and stromal ER-*β* cell activation [185, 186]. Genistein has suppressive effects on E2-stimulated ELT-3 cell proliferation in ELT-3 cells (uterine leiomyoma cell line) through PPAR*γ* activation [187].

*(2) Cervical Cancer*. Genistein inhibited the feasibility of HeLa cells by stimulating apoptosis, activating ER stress, and upregulating CHOP expression and glucose-regulated protein 78 in a dose-dependent manner [188]. Genistein improves intracellular p53 stability in cervical cancerous cells (HeLa) by interfering with the interaction of p53 and APE1. Moreover, it was also evaluated that interaction between p53 and APE1 plays an important role in degrading p53 and is dependent on the redox domain of APE1 [189, 190].

In cervical cancer HeLa cells, the combination of genistein (25 *μ*M) with cisplatin (250 nM) resulted in higher growth inhibition. Genistein boosts up the antitumor activity in cisplatin and inhibits the expression of p-p70S6K1, NF-*κ*B, p-4E-BP1, p-mTOR, and p-Akt. Through inhibition of Akt/mTOR and NF-*κ*B pathways, genistein can enhance cisplatin activity [191]. Genistein exposure to HeLa cells can significantly inhibit the time- and dose-dependent growth mediated by cell cycle arrest and apoptosis at the G2/M phase. Furthermore, it stimulated migration inhibition by modulating the expression of MMP-9 and tissue inhibitor of metalloproteinase- (TIMP-) 1 in a time-dependent manner. To prevent invasion, cancer cell growth, and metastasis, genistein can be an important antineoplastic agent [192]. Genistein significantly improves LDH release and lymphocyte proliferation in the TC-1 tumor cell line of adult female C57BL/6J mice. In addition, the treatment of genistein can cause a significant increase in interferon *γ* (IFN-*γ*). Furthermore, in tumor models, significant therapeutic effects were achieved through the treatment when compared to the control group. On tumor growth, genistein has a significant effect that may contribute to its effect on cytolytic activity, lymphocyte proliferation, and production of IFN-*γ* [193].

Genistein (20 and 60 *μ*M) significantly decreases the growth of CaSki and HeLa cells at various concentrations [194]. It reduced ERK1/2 and phosphorylation of the Akt pathway and stimulated JNK and p38 mitogen-activated protein kinase (MAPK). Furthermore, genistein downregulation of ERK1/2 activity increases cell growth reduction and p38 MAPK activity inhibited from genistein-mediated growth reduction [194]. The combination of gamma irradiation and genistein treatment can inhibit the G1 phase and increase the G2 phase up to 56%. They also increase p21, cdc2-Tyr15-p, and p53 expression by supporting G2/M arrest. Generally, apoptosis signaling was activated through the following processes: Bax upregulation, activation of caspase-3 and caspase-8, cytochrome c release, and Bcl-2 downregulation in the treatment of irradiation and genistein. Cotreatment inhibited the transcripts of E7, E6∗II, and E6∗I.

In cervical cancer cells, genistein also induced irradiation intracellular ROS and inhibited cotreatment that stimulated apoptosis by antioxidant N-acetylcysteine [195]. Genistein also increases gamma irradiation and ROS suggesting apoptosis in cervical cancer cells. COX-2 expression increased by gamma irradiation, while the combination of gamma irradiation and genistein prevented prostaglandin E2 (PGE2) production and expression of irradiation-induced COX-2. A combination of gamma irradiation and genistein can prevent proliferation by G2/M arrest and stimulated apoptosis through ROS modulation in cancer CaSki cells [195]. Genistein (40 *μ*M) combined with ionizing radiation (IR) (4 Gy) on cervical cancer cells (HeLa) increases the apoptosis index, and in the G2/M phase, cell arrest was enhanced. After IR (4 Gy), the expression of survivin mRNA increased, while it considerably reduced after combined treatment. In cervical cancer cells (HeLa), genistein improved radiosensitivity and this mechanism also includes the apoptosis increase and inhibited the survivin expression and persistence of cell cycle arrest [196].

*(3) Ovarian Cancer*. In estrogen-responsive cancers, EMT is important to process in progression and migration which is activated through E2. As usual endocrine-disrupting chemicals (EDCs), nonylphenol (NP) and bisphenol A (BPA), can migrate estrogen-responsive cancers and promote EMT. As a result, BPA and E2 enhance the protein expression of cathepsin D, MMP-2, and vimentin but inhibited the protein E-cadherin expression through the ER signaling pathway signifying that BPA and E2 promote cell migration and EMT. Moreover, through BPA and E2, the increased expression of protein MMP-2, vimentin, and cathepsin D was reduced by the combined treatment of GEN. In a scratch assay, BPA, NP, and E2 enhance the migration capability of BG-1 cells through ER signaling but the case was opposite when cotreated with genistein. In protein expression of Smad3 and SnoN, E2, BPA, and NP upregulated SnoN, a negative regulator of the TGF-*β* signaling, and inhibited pSmad3 in the downstream pathway, a transcription factor of the TGF-*β* signaling pathway, which leads to a conclusion that BPA, E2, and NP downregulated the TGF-*β* signaling in the procedure of migration of BG-1 cells and induction of EMT via ER signaling. By estrogenic chemicals, the combined treatment of genistein upturned downregulation of the TGF-*β* signaling. Conclusively, genistein migrated capacities of ovarian cancer BG-1 cells and suppressed EMT through BPA, E2, and NP and inhibited TGF-*β* signaling [197].

7-Difluoromethoxyl-5,4′-di-n-octylgenistein (DFOG) is a synthetic genistein analogue that possesses anticancer activities in various cancers that also includes ovarian cancer. DFOG inhibits the abilities of OCSLCs (ovarian cancer stem-like cells) by downregulating the expression FOXM1 [198].

*(4) Testicular Cancer*. Genistein significantly suppressed the level of intratesticular testosterone (ITT), and in rats, it enhances the recovery of spermatogenesis handled with a chemotherapeutic drug [199]. On TM4 testis cells, genistein exerts the following time- and dose-dependent effects:

- Through lower concentrations, apoptosis is stimulated, and through higher concentrations, necrosis is induced
- Genistein enhances the enzymatic activity of caspase-3
- Genistein induction of necrosis and apoptosis was significantly downregulated through the caspase-3 inhibitor, Z-DEVD-FMK
- Without induction of apoptosis and activation of enzymatic activity, CPP32 sodium azide induced necrosis
- Induction of apoptosis through genistein was related to the activation of enzymatic activity of CPP32 in cells [200, 201]

*(5) Bone Cancer*. On osteosarcoma cells (MG-63), genistein has antiproliferative effects through enhancement in peroxisome expression and cell growth inhibition [202]. Human tumor model osteosarcoma MNNG/HOS upregulates the antitumor activities of gemcitabine. The combined treatment of gemcitabine and genistein resulted in apoptosis induction and growth inhibition through activation of Akt and by downregulating the activity of NF-*κ*B in osteosarcoma cells. Moreover, when genistein was replaced by the NF-*κ*B or PI3K/Akt pathway inhibitor, synergetic effects were observed. *In vivo*, both enhance tumor growth inhibition through the downregulation of Akt activation and NF-*κ*B activity [203]. In left ventricle female mice, BALB/c-nu/nu was injected with antireceptor human breast cancer (MDA-MB-231) cells for the formation of osteolytic bone metastases. The administration of genistein (10 mg/kg per day) noticeably reduces the volume and number of osteolytic bone metastases. Moreover, histomorphometric analysis proved that genistein increased the trabecular thickness, number, and area and reduced trabecular separation [204]. Genistein reversed cancer resistance to gemcitabine in osteosarcoma (U2OS and MG-63) cell lines through inhibition of NF-*κ*B activity and Akt suppression. By reversing the Akt/NF-*κ*B pathway, the combination of genistein and gemcitabine upregulates antitumorous efficacy [205]. Oral pretreatment of genistein 20 mg/kg in DMBA-treated animals for 5 days considerably decreases the chromosomal abnormalities and frequency of micronucleus formation and also reversed the condition of biochemical variables [206–208].

**Figure 4:**
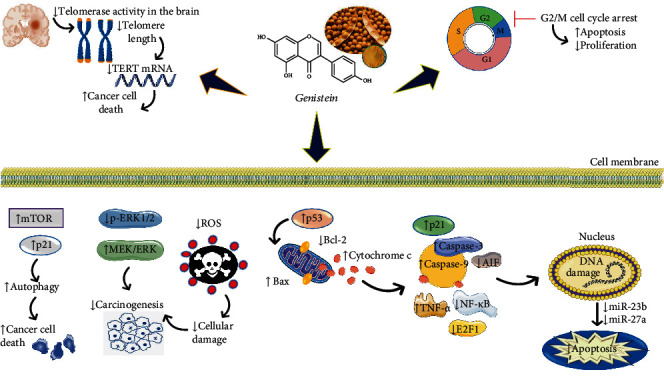
Diagram with the main molecular targets and signaling pathways of genistein as a potential anticancer agent. Abbreviations and symbols: ↑: increase; ↓: decrease; Bcl-2: B-cell lymphoma 2; mTOR: mechanistic target of rapamycin; p21: cyclin-dependent kinase inhibitor; p-ERK1/2: Ras-dependent extracellular signal-regulated kinase; MEK: mitogen-activated protein kinase kinase; ERK1: extracellular signal-regulated kinase; ROS: reactive oxygen species; AIF: apoptosis-inducing factor; TNF-*α*: tumor necrosis factor *α*; E2F1: transcription factor 1.

*(6) Skin Cancer*. Skin cancer is the most common form of cancer and is easy to treat if discovered early [209]. In the cell line of C57BL/6J mice (B164A5 melanoma), isoflavonoid genistein reduces tumor weight and volume. It inhibited the degree of erythema and quantity of melanin in the direct percentage to the number of days [210]. In LiBr cells, administration of genistein (40 *μ*M) restrained Livin gene (87.94%) expression and stimulated apoptosis for 48 hours in both the early and late (27.87 ± 5.38% and 11.87 ± 3.86%, respectively) phases. It significantly inhibited caspase-3 protein expression and reduces cell proliferation. Moreover, it also stimulates LiBr cell apoptosis, restrains cycles of cell generation and cell proliferation, and inhibits Livin gene expression [119]. The combination of genistein and ceramide (C6) stimulated significant Akt inhibition, caspase-3 cleavage, JNK activation, and cytochrome c release [211]. Genistein (50 *μ*M) downregulates the uveal melanoma C918 cell survival in humans *in vitro* by decreasing the vasculogenic mimicry in the section of tumor tissues. In C918 cells, it significantly inhibited protein expression and VE-cadherin mRNA [212, 213]. The coadministration of cisplatin and genistein can significantly inhibit Bcl-xL and Bcl-2 protein and enhanced Apaf-1 protein expression [214–216]. The most important molecular targets and signaling pathways of genistein in cancer cells are summarized in Figure 4.

### 4.4. Anti-Inflammatory Activity

Anti-inflammatory effects of ethanol extracts from seeds *Lupinus albus* L. and *Lupinus angustifolius* L. were measured using SKH-1 hairless mice [217]. Inflammation was induced as the ear edema by topical application of TPA 2 *μ*g/ear dissolved in 20 *μ*L acetone. The dose of extracts was 100 *μ*L (50 mg/mL). There was the middle activity of extracts from germinated seeds of both species but no activity for ungerminated seeds.

A series of works [39, 40] demonstrate the anti-inflammatory activity of several modified polyamide membranes modified with genistein. Proinflammatory factors such as TNF-*α*, interleukin- (IL-) 1*β*, and IL-6 in the human blood were stimulated to express by lipopolysaccharide (LPS). The modified genistein forms demonstrated higher activity against TNF-*α* and IL-1*β* and less active suppression against IL-6. Also, it showed that modified polyamide membranes with genistein (5-10%) may decrease platelet adhesion in the LDH test with scanning electron microscopy and hydrophilicity of these complexes was investigated by contact angles of water on the membrane surfaces using a commercial contact angle meter [40].

In the case of treatment, chronic rhinosinusitis genistein may decrease the nasal polyp fibroblast survivability and growth rate in a dose-depend manner [218]. Genistein (5-500 *μ*M) and phytic acid were tested on the fibroblast cells. Inhibition of expression of histone H3 and induction of apoptosis of cells were detected by modulating the expression of Bcl-2, Bax, and caspase-8 activity.

### 4.5. Antibacterial and Antiviral Activity

Genistein antibacterial activity was investigated using *Bacillus subtilis*, *Enterococcus faecalis*, *Escherichia coli*, *Salmonella typhimurium*, *Shigella sonnei*, *Pseudomonas aeruginosa*, and *Staphylococcus aureus* by the agar disk diffusion method [219–221]. The most sensitive culture to all genistein complexes and pure genistein was *Bacillus subtilis* (15-17 mm of zone inhibition). Against this gram-positive culture, the most active was GEN-HPGCD (genistein-hydroxypropyl-gamma-cyclodextrin, ratio 1 : 1) [48]. At the form of genistein-polyurethane microstructures against 7 bacterial cultures (*S*. *aureus*, *E*. *coli*, *P*. *aeruginosa*, *Salmonella enteritidis*, *B*. *cereus*, *B*. *subtilis*, and *Candida albicans*) by the dilution method, it was not active in comparison with pure genistein. Genistein (10 mM) was active against *B*. *subtilis*, *B*. *cereus*, and *C*. *albicans*.

Viral agents can generate persistent infections in humans, sometimes with severe complications, and therefore, pharmacological and alternative therapies are needed [222, 223]. Rotavirus was inhibited by genistein in the MA104, HT-29, SW620, and Caco-2 cells [224]. Genistein did not affect rotavirus binding and entry but inhibited replication and synthesis of viral protein.

### 4.6. Antidiabetes and Effects on Lipid Metabolism

The effect of genistein on type 2 diabetes mellitus (T2DM) was described [225]. One of the targets to treat this type of diabetes may be phosphoenolpyruvate carboxykinase (PEPCK). Its isoform, cytosolic PEPCK (PEPCK-C), may be regulated due to liver gene expression. It may be recognized by AMPK, an enzyme which regulates the expression of PEPCK. Genistein inhibited PEPCK-C expression by phosphorylation states of AMPK, MEK1/2, ERK1/2, and CRTC2; also, genistein decreased glucose levels [225]

One possible pathway to progress diabetes may be dicarbonyl stress such as methylglyoxal (MGO). Wang et al. [226] demonstrated the possibility of genistein trap MGO *in vivo*. Female C57BL/6J mice were treated with genistein (400 mg/kg body weight in DMSO in the acute study and 130 mg/kg body weight in DMSO oral in the chronic study) and MGO (1.0 g/kg body weight in water after treatment by genistein in the acute study and 0.96% MGO for drinking during the month before genistein in the chronic study). Urinary metabolites were analyzed by liquid chromatography-mass spectrometry (LC-MS). The results showed that *in vivo* genistein forms with several MGO metabolites, but only one metabolite is formed among the reaction A ring of GEN (DGEN, orobol, 5-OH-equol, and 6′-OH-DMA) and MGO. The other five metabolites form as microbial derivatives. Therefore, genistein may trap MGO to prevent hyperglycemia and diabetes.

Using a mouse culture model C_2_C1_2_ myogenic cell line [227], glucose transport and oxidation of fatty acids by measuring the radioactivity of 2-deoxy-D-[1,2-^3^H]-glucose and [9,10-^3^H]palmitic acid, respectively, were investigated. The dose of genistein was 0.1, 1, and 50 *μ*M. Also, the effect on the expression of protein kinase B by genistein was measured using western blotting. In the dose 1 *μ*M, genistein increased the levels of phosphorylation of PKB, one of the major enzymes of the insulin pathway. Also, by microarray analysis, increasing levels of expression of 11 genes were detected (insulin receptor family, PI3 kinase pathway, MAPK kinase pathway, insulin pathway, carbohydrate metabolism, lipid metabolism, transcription factors, and regulatory genes of the cell cycle or differentiation) by genistein (1 *μ*M). The reduction of gene expression was detected for nck1 (noncatalytic region of tyrosine kinase adaptor protein 1) after 4 hours of incubation, including the reduced expression of cbl (Casitas B-lineage lymphoma), gck (glucokinase), and sorbs1 (sorbin and SH3 domain containing 1) and the increased expression of akt1 (serine/threonine-protein kinase 1) genes after 24 hours of incubation.

Genistein may decrease the level of leptin in adipocytes [228, 229]. Zanella et al. [230] compared several groups of male C57BL/6J mice that had a low-fat diet (LFD). Mice were treated with pure genistein (5 mg/kg/day) and E2 (5 *μ*g/kg/day). Body fat deposition and gene expression profiles were measured. Also, there were two groups: those fed by 8.5% soy-supplemented LFD (SS-LFD) and those fed by soy-free LFD (SF-LFD). In the results, the total fat mass of mice was higher in the SF-LFD group. Glucose metabolism and insulin sensitivity were not altered in these mice. There were no significant differences in the total lean mass and total body weight. In comparison to genistein as an estrogen active agent, E2 reduced weight fat pads in the mice with SF-LFD. Also, among 11 genes, including adipocyte metabolism, genistein upregulated 6. The rest of them was PPAR*γ*.

It was found that genistein and genistein 4′,7-*O*-dioleate may protect low-density lipoproteins from oxidation, one of the factors of atherosclerosis [231]. This may be used in the treatment of atherosclerosis. These compounds are used to treat adult female rhesus (*Macaca mulatta*) monkeys by subcutaneous injection of genistein (24 mg) and genistein 4′,7-*O*-dioleate (71 mg) or oral administration (same doses). All metabolites were detected in the plasma by time-resolved fluoroimmunoassay (TRFIA). In the results, the plasma concentration of genistein 4′,7-*O*-dioleate fatty acid ester was 7.5 nM after 8 hours and 12 nmol/L after 24 hours at the subcutaneous administration. In one monkey, orally administered free genistein had probably become esterified (24 h value 6.1 nM), but oral genistein dioleate intake did not result in elevated levels of esterified genistein. These results demonstrated the ability to detect acid ester conjugates at the subcutaneous treatment but not oral treatment.

Kaamanen et al. [232] found that in the human blood samples, genistein may protect low-density lipoproteins into the arterial intima. Genistein inhibits methylglyoxal/lysine glycation-induced DNA strand breakage and ROS generation in the presence and absence of Cu^2+^ [233].

*In vivo* antidiabetes was also shown in old male and female ob/ob and lean C57BL/6J mice with a genistein diet (600 mg genistein/kg food) [234, 235] and in male Sprague-Dawley rats (diet with 0.25 mg/kg/day/rat genistein and drinking water 20% fructose) [236].

## 5. Genistein in Clinical Studies

### 5.1. Anticancer and Cytotoxic Activity

#### 5.1.1. Breast Cancer

One of the first clinical trials studying the effects of soy supplementation on the proliferation rate of premenopausal women was carried out by McMichael-Phillips et al. [238]. The serum levels of genistein and daidzein were increased in the soy group after 14 days of 60 g soy supplementation (45 mg isoflavones). This study concluded that short-term dietary soy stimulated breast proliferation and progesterone receptor expression.

The results obtained by Hargreaves et al. [239] with the same treatment suggested that soy isoflavones had weak estrogenic activity as pS2 expression increased in nipple aspirate of those patients with soy supplementation, but no effects were observed on breast epithelial cell proliferation, estrogen and progesterone receptor status, cell death, cell cycle progression, or Bcl-2 expression. The clinical trials carried out in the following years were focused on the levels of isoflavones in plasma and their urinary excretion, as well as the production of equol, a daidzein metabolite of gut bacteria in patients with or without breast cancer [240–242].

Bolca et al. [243] performed a very interesting clinical trial about the disposition of soy isoflavones in normal breast tissue. In this study, the authors evaluated the potential health effects of isoflavone consumption on normal breast tissue, investigating the isoflavone concentrations, metabolites, and biodistribution compared to estradiol exposure. The blood and the normal breast tissue were collected during esthetic breast reduction. After analysis with LC-MS, they demonstrated that after intake of soy milk and soy supplements (with significant amounts of genistein), isoflavones reach exposure levels in breast tissue at which potential health effects may occur [243]. This clinical trial represented a major step forward in the study of isoflavones and, therefore, genistein in breast tissue modulation due to the knowledge of the biodistribution of the phytoestrogens in this tissue.

Another study observed an inverse association between plasma isoflavones like daidzein and genistein and fibroadenoma risk (the most common benign breast condition, which could develop into breast carcinoma), suggesting that higher intake of soy foods may lower the risk of fibroadenomas and, therefore, breast cancer [244].

The most recent studies suggest that the administration of genistein did not reduce breast epithelial proliferation, but it could increase the risk of breast cancer. Khan et al. [245] performed a trial involving 98 women who underwent fine-needle aspiration. The results showed that most of the genes analyzed presented significant increases in their expression in the 6-month soy-treated group, suggesting a lack of efficacy for breast cancer prevention and a possible adverse effect in premenopausal women [245].

Shike et al. [246] evaluated the gene expression profile in early-stage breast cancer in tumor tissue of women before and after treatment with a soy protein supplement and placebo. The authors reported that gene expression associated with soy intake and high plasma genistein produced the overexpression of genes involved in the cell cycle and proliferation [246].

#### 5.1.2. Endometrial Cancer

A clinical trial was carried out to evaluate the effects of genistein aglycone in reducing endometrial hyperplasia, which is not cancer, but it could be the beginning of neoplasia [9]. In this study, Bitto et al. [9] suggested that genistein aglycone could be useful for the management of endometrial hyperplasia without atypia in women that cannot be treated with progestin, the most common treatment for this pathology. The genistein treatment produced a decrease in the endometrial thickness and the immunohistochemical staining for the ER-*α* and the progesterone receptor, as well as an increase of ER-*β*1 staining, associated with complete regression of bleeding [9].

#### 5.1.3. Prostate Cancer

Most epidemiological studies have shown an inverse association between soy consumption and the risk of prostate cancer in the Asian population, as soy is the main source of protein in the Asian diet [247–249].

Urban et al. [250] revealed an important issue: soy protein beverage (containing significant levels of genistein and daidzein) supplementation in men reduced serum cholesterol levels but not the prostate cancer biomarkers prostate-specific antigen (PSA) and p105erB-2 [250]; the same result was obtained by other authors years later [251, 252].

Other studies suggested significant changes in PSA and/or in blood cholesterol in soy-treated patients with prostate cancer, with a favorable influence [253–255]. In a more recent study, Lazarevic et al. [177] demonstrated that genistein could reduce the expression of several biomarkers related to prostate cancer prediction and progression such as androgen-related biomarkers (KLK4) and cell cycle-related genes (p27Kip1), supporting genistein as a chemopreventive agent.

A study carried out in Australia evaluated the effects of red clover-derived dietary isoflavones (genistein, daidzein, formononetin, and biochanin A) in patients with prostate cancer [256]. Although other parameters like PSA or testosterone serum levels did not raise significant changes between groups, they found that the apoptosis index was significantly higher in isoflavone-treated patients, suggesting that isoflavones may halt the progression of prostate cancer via apoptosis induction in low- to moderate-grade tumors [256].

Another relevant study showed that genistein did not cause genetic damage in subjects with prostate cancer treated with a purified soy unconjugated isoflavone mixture composed of genistein, an important finding to use phytoestrogens like genistein to prevent or treat prostate cancer [257].

Rannikko et al. [258] found that, after phytoestrogen consumption, the concentrations of genistein and daidzein in prostate cancer patients were increased in prostate tissue, suggesting that the biological functions of these isoflavones could be done *in situ* in the prostate. Years later, other authors confirmed that prostate tissue may have the ability to concentrate dietary soy isoflavones to potentially increase anticarcinogenic levels [259]. Guy et al. [260] found that these phytoestrogens could be glucuronides in the prostate tissue.

Prostaglandins are known to be stimulators of prostate cancer growth. Swami et al. [261] demonstrated that in prostate cancer patients, genistein consumption provoked a decrease in COX-2 and an increase in p21 gene expression in prostate tissue obtained by prostatectomy, suggesting that genistein consumption could be beneficial in prostate cancer chemoprevention and/or treatment through the inhibition of the prostaglandin pathway.

Another study related the MMP-2 transcript levels in prostate cancer patients treated or not treated with genistein. Results showed that the MMP-2 transcript level in normal prostate epithelial cells from prostate cancer-containing tissue was significantly higher in the control group than in the genistein-treated group [262].

A study carried out by Bilir et al. [263] provided new knowledge about the genistein supplementation effects on genome-wide DNA methylation and gene expression in patients with localized prostate cancer using microarrays. Whole-genome methylation and expression profiling identified differentially methylated sites and expressed genes between groups, placebo and genistein. Differentially regulated genes were related to developmental processes, stem cell markers, proliferation, and transcriptional regulation, suggesting the importance of genistein in gene expression changes in prostate cancer and the effects of genistein on molecular pathways involved in prostate tumorigenesis [263].

The most recent clinical trial was focused on examining the difference between short- and long-term genistein treatment effects, comparing the US and Chinese cohorts [264]. The authors demonstrated that treatment with genistein selectively targeted genes in at-risk prostate tissue that regulate prostate cell motility. Moreover, they observed that the pharmacologic target of genistein was downregulated in Chinese men, who experience lifetime exposure to dietary genistein, suggesting that biomarker expression may be modified as a function of treatment time [264].

#### 5.1.4. Urinary Tract Cancer

The effects of genistein administration in patients with kidney neoplasm were evaluated by Yasuda et al. [265], suggesting that this phytoestrogen could enhance the superoxide generation after cisplatin treatment due to its property as a protein kinase inhibitor.

Messing et al. [160] published a study to evaluate the effects of genistein consumption (as the purified soy extract G-2535) and the amount of phosphorylated epidermal growth factor receptor (p-EGFR), due to the potential mechanism of genistein to inhibit this receptor phosphorylation and, therefore, to inhibit cell proliferation. In this clinical trial, patients were treated with a placebo or two different amounts of genistein (300 or 600 mg/day) and the results suggested that genistein displayed a possible bimodal effect, more effective at the lower dose reducing the EGFR phosphorylation in bladder cancer tissue observed with immunohistochemistry. No differences were observed in the normal bladder epithelium as well as in the tumor tissue staining between treatment groups for COX-2, Ki-67, activated caspase-3, Akt, p-Akt, MAPK, or p-MAPK. The authors recommend further evaluation of the effects of genistein in combination with other agents such as other ER modifiers or chemotherapeutic agents [160].

#### 5.1.5. Pancreatic Cancer

Genistein can inactivate the Akt and NF-*κ*B signaling pathway, which is frequently deregulated in pancreatic cancer contributing to cell growth, metastasis, and chemoresistance. A phase II study carried out by El-Rayes et al. [266] demonstrated that in the case of patients with advanced pancreatic cancer, the addition of soy isoflavones to gemcitabine and erlotinib did not increase the survival of these patients. Löhr et al. [267] published a phase I clinical trial with AXP107-11, a multicomponent crystalline form of genistein, to assess its safety, maximum tolerated dose, and pharmacokinetics in combination with gemcitabine in treatment-naïve patients with inoperable pancreatic carcinoma. The results showed a favorable pharmacokinetic profile without signs of toxicity, suggesting further studies with this genistein analogue in pancreatic patients [267].

#### 5.1.6. Colon Cancer

In a trial carried out by Adams et al. [268], a 12-month randomized intervention was conducted in men and women aged 50-80 years with recently diagnosed adenomatous polyps. In this study, the researchers investigated the cell proliferation with Ki-67 immunohistostaining in patients treated with relatively high doses of soy isoflavones, including genistein. In this case, they concluded that soy protein supplementation containing isoflavones did not diminish colorectal epithelial cell proliferation or the average height of proliferating cells in the cecum and rectum and enhanced cell proliferation measures in the sigmoid colon [268].

### 5.2. Climacteric Symptoms

Hot flushes are one of the main symptoms of menopause. In a recent study carried out in Italy, the authors observed that genistein could reduce hot flushes decreasing the circulating levels of visfatin, an inflammatory adipokine secreted by visceral fat [269]. Other similar studies demonstrated that the treatment with a phytocomplex containing isoflavones was able to counter symptoms of the climacteric syndrome, such as hot flushes, insomnia, and depression [270]. Another study evaluated the effects of a single daily dose of 30 mg of synthetic genistein in healthy postmenopausal women, demonstrating that this treatment could reduce the hot flush frequency and duration [271].

Bone mass loss is a secondary effect of menopause, and genistein could have a preventive role against this process [272]. In the last years, some studies have shown that genistein could have a positive effect on bone health, alone or in combination with vitamins [273, 274].

A large study involving 389 women and 3-year genistein treatment found that genistein aglycone plus calcium, vitamin D_3_, and a healthy diet provoked positive effects on some cardiovascular risk factors and homocysteine levels, an independent risk factor for coronary artery disease, in a cohort of postmenopausal women with low bone mass [275].

Another study suggests that phytoestrogens like genistein could decrease bone turnover, preventing osteoporosis [276]. However, other clinical trials did not find significant results in improving bone density [277–279], so there is a controversy about this ability.

### 5.3. Diabetes and Lipid Metabolism

The effects of genistein in diabetic patients have been studied in a large study carried out in Italy by Squadrito et al. [280]. In this clinical trial, 120 postmenopausal women with metabolic syndrome participated in the study (*n* = 60 placebo and *n* = 60 54 mg genistein daily for 1 year). The results showed that genistein was able to reduce significantly the risk of diabetes [280].

Other authors have suggested a possible protective effect of antioxidants (including genistein) on retinal cells in preretinopathic diabetes patients [281]. In contrast, one previous study performed in normal-weight postmenopausal women did not find any significant improvement in metabolic parameters when a high-dose isoflavone supplement was given [282], suggesting that the beneficial effects of genistein could be observable in obese and insulin-resistant patients.

Lipid metabolism could be a possible target for genistein. Okamura et al. [276] performed a clinical trial to study the effects of phytoestrogens like genistein on lipid metabolism. The results suggested that phytoestrogens have beneficial effects on lipid metabolism in postmenopausal women through an increase of HDL and apolipoprotein A-1 and a decrease in LDL and apolipoprotein B [276].

### 5.4. Depression and Neurodegenerative Diseases

Neurodegenerative diseases refer to the fact that certain areas of the brain, spinal cord, or peripheral nerves no longer function normally, which results in the appearance of local dysfunctions of dying neurons [283]. Syndromes associated with neurodegenerative disorders can affect thinking, ability to move, endurance, sensations, coordination, and self-control.

In a recent study, a group of Alzheimer's patients was treated with 100 mg/day of soy isoflavones including mainly daidzein and genistein (85%) [284]. Plasma isoflavone levels increased in participants treated with soy isoflavones. After six months of treatment with soy isoflavones, results did not show an improvement in cognition capacities in older men and women with Alzheimer's disease. However, patients with higher equol plasma levels presented speeded dexterity and verbal fluency, suggesting that more studies are needed to examine the role of isoflavone metabolism, for example, the metabolization of these soy isoflavones to equol to clarify their cognitive effects

In a clinical trial carried out by de Ruijter et al. [285] with Sanfilippo disease patients, genistein at a concentration of 10 mg/kg/day reduces the urinary excretion of glycosaminoglycan and the plasma heparan sulfate concentration, substances related to the Sanfilippo disease. However, the authors concluded that this reduction with small and higher doses of genistein might be more effective in these patients [285].

Genistein significantly ameliorates bone loss during menopause.

The most important key findings of human clinical trials are summarized in Table 2.

## 6. Toxicological Aspects

Genistein can interfere with thyroid regulation. Soy foods may increase the risk of hypothyroidism in people with impaired thyroid function, and they may develop goiter and autoimmune thyroid disease. This risk increases even more when the individual's iodine intake is low. Soy genistein has been found to inhibit the activity of an enzyme called thyroid peroxidase, which is needed for thyroid hormone synthesis. Therefore, there is a risk of hypothyroidism when consuming too much soy protein. Soy products also interfere with the absorption of levothyroxine (L-thyroxine), a drug used to treat thyroid hormone deficiency [286].

Genistein can cause testosterone imbalance. One study was performed on 12 male subjects who consumed 56 g of soy protein daily for four weeks. As a result, their serum testosterone levels decreased by 19%. Although the data were inconsistent, it was found that genistein decreased serum testosterone levels in healthy men [287].

Genistein can cause hypersensitivity (allergy). Soy products can cause allergies or hypersensitivity in children and adults. Often, soy allergy starts in childhood, in reaction to soy products that can cause allergies or hypersensitivity in children and adults. Often, soy allergy begins in childhood with a reaction to soy-based infant formulas. However, most children overcome soy allergy. In general, soy allergy is uncomfortable but not severe. An allergic reaction to soy is rarely frightening or lethal. Symptoms of soy allergy may include paresthesias, eczema or itchy skin, wheezing, diarrhea, stomach pain, vomiting, and redness of the skin [288].

Genistein can increase the risk of cancer proliferation, especially estrogen-dependent breast cancer, because soy genistein tends to have estrogenic effects. According to animal studies, genistein can disrupt the cell cycle and trigger tumor development. It works by triggering estrogen receptors. In contrast, human studies show an inverse relationship between cancer and genistein, reducing the incidence and mortality rate caused by breast cancer. This may be due to the antiestrogenic effect of phytoestrogens [289].

According to the US FDA, daily consumption of 25 g of soy seems safe without triggering side effects. This amount of soy could also help lower cholesterol levels. It is also believed that the intake of 50 g of soy protein per day could help prevent heart disease, diabetes, and estrogen-dependent cancers. However, more research is needed. There is limited information on the excess intake of soy. But no more than 25 grams a day of soy is recommended [290].

## 7. Discussion

The strength of our study is the inclusion of many meta-analyses on the pharmacological effects of genistein. From their analysis, it was possible to highlight the molecular targets on which genistein acts, thus opening new anticancer therapeutic perspectives. Also, very recent studies have shown that genistein nanoformulations increase its bioavailability, consequently an increased pharmacotherapeutic effect. Therefore, genistein can be considered an effective therapeutic adjuvant. Limitations of the paper are derived from the clinical pitfalls of effective therapeutic doses because translational medicine studies are insufficient.

Genistein has been used as the food isoflavone in soybean products. They are popular among Asian people as nutrient and food components, and there are data about reducing the risk of many diseases. There are a lot of data about anticancer activity against several types of cancer; it has hypolipidemic and antiatherosclerotic effects, used as an antidiabetic agent; it may decrease the risk of cardiovascular disease, may also induce angiogenesis, and may be used in the treatment of rheumatoid arthritis [11, 291–293]. The main mechanisms of activity of genistein are regulation of ERs and inhibition of protein tyrosine kinase, nuclear factor-*κ* B (NF-*κ*B), and topoisomerases I and II. Among the various pathologies, endocrine disorders are arousing increasing interest, and in this sense, the class of secondary metabolites that is most investigated is certainly that of the so-called phytoestrogens, which includes several polyphenol classes (flavones, isoflavones, coumestans, lignans, chalcones, and prenylflavonoids), of which the maximum representative is certainly isoflavones [294–296]. They are secondary metabolites found in the food commonly consumed by the East Asian population, so much so that chronic soybean consumption is directly correlated with some human diseases such as cardiovascular diseases, osteoporosis, and certain types of cancer found in China and Japan in comparison to those of western countries [15].

Phytoestrogens present in plants do not act as hormones; they act as phytoalexins, plant-derived low-molecular compounds synthesized and accumulated in response to abiotic and biotic stressors, with antimicrobial and antioxidant properties [295]. Boonpawa et al. [297] showed the estrogen effects of genistein and the genistin metabolite pathway using a physiologically based kinetic (PBK) model. It was found that the main metabolite is genistein-7-*O*-glucuronide. In the blood, the low concentration of free aglycones (about 0.5–17% of total plasma genistein using 0.0003–77 *μ*M at oral doses ranging from 0.01 to 50 mg/kg genistein) was found. The estrogen effect of the mixture of phytoestrogen genistein and two mycotoxins such as zearalenone from *Fusarium* toxin and alternariol from *Alternaria* spp. was observed [298]. The mechanism of genistein activity was established using porcine granulosa cells (3 types of cells: unseparated granulosa cells and antral and mural cells isolated from pig ovaries) [299–301]. It was found that genistein has an affinity to both ERs (ER-*α* and ER-*β*) with higher activity to ER-*β*. Also, it is effective on the secretion of progesterone (P4) and E2 [302].

Calcium homeostasis is one of the causes by which postmenopausal hormone replacement therapy is a risk factor to suffer myocardial infarction, stroke [303], and pulmonary embolism, which are mediated by calcium-induced signaling [304]. Soy isoflavones could be potential alternatives to hormone replacement therapy without these adverse effects, as dietary calcium in combination with soy isoflavones (mainly daidzein and genistein) has not been associated with increased risk of myocardial infarction and venous thromboembolism [305]. Other authors have reported recently an amelioration of ischemic cardiomyopathy by isoflavone supplementation via upregulation of Nrf2 and superoxide dismutase, improving the antioxidant capacity of isoflavone-treated patients [306]. Recent studies have related the genistein intake with endothelial function in postmenopausal women with metabolic syndrome, suggesting that six or twelve months of treatment with genistein effectively improved brachial artery flow-mediated vasodilation and other markers associated with diabetes and cardiovascular disease [280, 307]. Depression is another characteristic of postmenopausal estrogen decline in women; therefore, the treatment with phytoestrogens like genistein could provide a solution to eliminating these symptoms. Characterized by a negative emotional state, depression is a complex problem and characteristic of neurodegeneration [308] [309].

From physiological reactions such as insomnia or lack of appetite to irritable behavior, the symptoms of this condition are varied and are not always easy to treat [303]. There are some studies in the last years suggesting that the consumption of soy isoflavones, more concretely genistein, improved the quality of life in postmenopausal women, increasing health status and life satisfaction and ameliorating depression perception. The health properties of phytoestrogens seem to go beyond their estrogen-like activity. Many studies have demonstrated that they, acting on other target molecules and signaling pathways, exert several health effects such as antiandrogenic properties, antioxidant action, cell cycle and differentiation inhibition, antiangiogenic properties, and modulation of the activity or expression of steroidogenic enzymes [310]

The antioxidant activity of genistein in *in vitro* and *in vivo* studies has been widely demonstrated. The results of clinical studies on genistein antioxidant activity evaluation have been clear, suggesting that this antioxidant activity could be the most reliable effect of this phytoestrogen. For example, Li and Zhang [306] published that an isoflavone extract composed of 55% genistein ameliorated ischemic cardiomyopathy patients by enhancing their antioxidant capacities via Nrf2 upregulation. Other clinical trials have been related to the antioxidant and anti-inflammatory activities of genistein with better lung function and asthma control. In addition to this, antioxidant treatment, including genistein, may have a protective effect on retinal cells in preretinopathic diabetes patients [281].

Several clinical trials are reporting the relationship between genistein and breast cancer incidence and/or treatment efficacy. The implication of estrogens in breast cancer risk and the nature of genistein as a phytoestrogen make genistein an interesting factor to consider in breast cancer.

The beneficial effect of genistein has been indicated on the inflammatory state in a study with nonalcoholic fatty liver patients, reducing oxidative and inflammatory indices and insulin resistance [311]. Another clinical study suggested that isoflavone supplements, both with low and high doses of genistein, induced anti-inflammatory gene expression in equol-producing postmenopausal women [312]. Moreover, a positive effect of isoflavone-rich foods, with different amounts of genistein, was reported in the inflammatory and nutritional status in hemodialysis patients with underlying systemic inflammation [313].

Cancer is one of the leading causes of death worldwide, and the number of cancers is on the rise [314]. The main goals in cancer therapy include the removal of the primary tumor, the prevention of metastases, the improvement of survival, and the quality of life of patients. Due to the toxicity of conventional treatments, the researchers sought to find a secondary compound that would increase the anticancer potential of primary therapy but would reduce toxicity [315].

Thus, the researchers studied the role of genistein in the treatment/prevention of cancer. Genistein therapy has been shown to inhibit inflammation, angiogenesis, and metastasis in many types of tumors. Remarkable benefits of this therapy have been observed in combination with radiotherapy [8].

## 8. Overall Conclusions and Future Perspectives

Genistein exerts several biological activities. As a phytoestrogen, in mammalians, it acts as an estrogen agonist or antagonist. However, the health properties of genistein seem to go beyond their estrogen-like activity. The reported preclinical pharmacological activities of genistein are many, such as antioxidant, anti-inflammatory, and antimicrobial activities, angiogenesis and estrogen effects, and pharmacological activities on diabetes and lipid metabolism. However, the most studied activities are anticancer and cytotoxic activities. In human cancer, genistein stimulated the downregulation of CIP2A, significantly reduced the cell number, caused cell arrest, and also upregulated the protein and mRNA expressions of TNFR-1. It also activated p53 protein and caspase-3 and caspase-10. Moreover, genistein suppressed the activation of HSC, decreased the expression in *α*-SMA and accumulation of the collagen matrix, and elevated serum ALT and AST levels. Furthermore, genistein also downregulated TGF-*β* expression and stimulated TGF-*β*/Smad signaling. The current review article highlights the preventive role of genistein bioactive compounds against various human cancers through multiple pathways. In various clinical studies on cancer types, the anticancer effects of genistein have been shown, with breast and prostate cancers presenting the most evidence. In the future, more clinical trials and research studies using genistein formulation with better bioavailability can open new horizons in the field of research.

## Figures and Tables

**Table 1:** *In vitro* preclinical studies regarding the anticancer molecular mechanisms of genistein.

Type of cancer		Cancer cell lines	Potential anticancer mechanisms	Ref
Brain tumors	Neuroblastoma	IMR-32 SK-N-BE2	↑Apoptosis, ↓cell viability, ↑Myd88, ↑Beclin 1, ↑LC3 II, ↑TLR4, ↑autophagy, ↓mTOR, ↓p62	[52]
		SK-N-SH	↑Cell cycle arrest at phase G2/M, ↓proliferation, ↑Akt, ↑CHD5, ↑p53, ↓neuroblastoma growth, ↓tumor microvessel formation, ↓DNMT3b, ↑ERE, ↑luciferase, ↑MEK	[53–55]
		SK-N-DZ	↑Apoptosis, ↑FasL, ↑TNFR-1, ↑TNF-*α*, ↑FADD, ↑caspase-8, ↓cell proliferation, ↑PARP, ↑DFF45 cleavage, ↑apoptosis	[57]
		SH-SY5Y (N-Myc nonamplified) SK-N-DZ (N-Myc amplified)	↑Suppression of survival and angiogenic pathways: cleavage of Bid to tBid, ↓hTERT, ↓VEGF, ↓NF-*κ*B, ↓c-IAP2, ↓MDR, ↓N-Myc, ↓FGF2, ↓p-Akt, ↑apoptosis	[58]
		SH-SY5Y SK-N-BE2	↑Apoptosis, ↓tumor weight, ↓volume, ↑Smac, ↑Bax, ↓Bcl-2, ↓BIRC, ↓AIF, ↓caspase-3, ↓VEGF, ↓FGF2, ↓NF-*κ*B	[59]
		SK-N-BE2	↑Apoptosis, ↑Bax, ↓Bcl-2, ↑mitochondrial release of cytochrome c, ↑AIF, ↑Smac, ↑Bax : Bcl-2 ratio, ↓N-Myc, ↑NF-*κ*B, ↑calpain, ↑caspase-3, ↑caspase-8, ↓SBDP	[60–62]
	Glioblastoma Medulloblastoma	Glioblastoma A172, KNS60, U251MG Medulloblastoma ONS76	↓Cell growth, ↑cell arrest at the G2/M phase, ↑DNA damage, ↓telomerase, ↑telomere shortening	[67]
	Pituitary cancer	Human prolactinoma cells	↑Apoptosis, ↑percentage of cells in phase G1, ↓DNA synthesis, ↓cell proliferation of cultured pituitary cells	[69]
		Mouse AtT-20 Rat anterior pituitary cells	↓Proliferation at the G0/G1 phase and G2/M phase, ↑apoptosis	[70]
Breast cancer		T47D MCF-7-C3	↓CIP2A, ↓E2F1, ↑apoptosis, ↑growth inhibition, ↑proteasomal degradation, ↑transcriptional suppression	[71]
		MCF-7 MDA-MB-231	↑ABCC1, ↑ABCG2, ↑apoptosis, ↓p-Akt, ↓IGF-1R, ↓Bcl-2-associated X protein-protein ratio	[72]
		BCSCs	↓CD44+/CD24-/ESA+, ↑PI3K/Akt, ↑MEK/ERK, ↑G2/M cell cycle arrest, ↑apoptosis, ↑BRCA1, ↑ATR complex, ↑DNA damage, ↓TNBC	[74]
		Hs578t MDA-MB-435	↑Apoptosis, ↓cell viability, ↑miR-23b	[75–79]
		MCF-7 T47D	↓IGF-1R-PI3K/Akt, ↓cell proliferation, ↓Bcl-2/Bax, ↓mRNA, ↑apoptosis, ↓Akt, ↓HOTAIR	[84, 86]
		MCF-7/Adr	Genistein combined with doxorubicin: ↑intracellular accumulation of doxorubicin, ↑apoptosis, ↑cell cycle arrest, ↓HER2/neu expression	[87]
		MCF-7	↑Ribose 5-phosphate, ↑6-phosphogluconate, ↑pentose phosphate pathway, ↓glutamine, ↓glucose uptake, ↓protein biosynthesis	[89–94]
Lung cancer		A549	↑Caspase-3/9, ↑apoptosis, ↓MET, ↑miR-27a	[97]
		A549 MRC-5	Radiosensitizing effect, ↑oxidative stress, ↓oxidative damage, ↑mRNA, ↑GSH, ↑Nrf2, ↑HO-1	[98]
		A549, NCI-H460 (H460), ABC-1	↑TSA, ↑histone or nonhistone protein acetylation, ↑histone H3/H4 acetylation, ↑expression of protein p300	[99]
		A549	Genistein combined with ATRA, ↓ICAM-1, Bcl-2, ↓MUC1, ↓Bcl-2, ↓Bax, ↓p-ERK1/2, ↓Cdk4, ↓Rb, ↓metastatic potential	[100]
		H446	↑Apoptosis, ↓cell proliferation, ↓FOXM1 protein, ↓proliferation	[101]
		A549	↓Cyclin D1, ↓Cdk4, ↑p15, ↑p21, ↑p27l, ↑Rb protein phosphorylation, ↑p53, ↑caspase-3, ↓TNFR-1, ↑apoptosis	[56]
Gastrointestinal cancers	Salivary gland cancer	SACC-83	↓Bax, ↓survivin, ↓Bcl-2, ↓protein tyrosine kinase, ↓cyclin D1, ↓cyclin B1, ↓Cdk4, ↓Cdk1, ↑G2/M cell cycle arrest	[106]
	Esophageal cancer	TE-2 (p53, wild) TE-1 (p53, mutant)	↑Radiosensitivity of cell lines, ↓p42/p44, ↑Akt/PKB, ↑poly(ADP-ribose) polymerase, ↑Bax, ↓Bcl-2	[111, 112]
	Gastric cancer	AGS MKN45	↓Chemoresistance to 5-FU and cisplatin, ↓ERK1/2, ↓ABCG2, ↓tumor mass, ↓CD44, ↓Gli1, ↓Gli1 siRNA	[113]
		BGC-823	↓p34(cdc2), ↑Tyr15, ↑G2/M cell cycle arrest, ↓COX-2, ↑apoptosis	[114]
		SGC-7901	↓Ser642, ↑PTEN, ↓CENPF, ↓KIF23, ↓KIF22, ↓KIF20A, ↓KIF11, ↓FOXM1, ↓cdc25B, ↓cyclin B, ↓Cdk1, ↑p27Kip1	[115–120]
	Liver cancers	PLC/PRF5	↑Apoptosis, ↑G2/M cell cycle arrest	[125]
		HepG2	↑MRP2 mRNA, ↑P-gp, ↓miR-379	[126]
		HepG2/C3A	Long term: ↑CYP1A Short term: ↓YP1A	[127, 128]
		BNL CL2, Huh7, HepG2, HA22T	↓MMP-9, ↓NF-*κ*B, ↓P-1, ↑AP-1, ↓JNK, ↓ERK, ↓NF-*κ*B, ↑phosphatidylinositol/ERK3-kinase/Akt	[129]
		SMMC-7721, HepG2, Bel-7402	↑*α*-Catenin, ↑E-cadherin, ↓vimentin, ↓N-cadherin, ↓mRNA, ↑EMT, ↑TGF-*β*, ↓autotaxin, ↓COX-2, ↓ABCA3, ↓CD154	[130]
	Pancreatic cancer	MIA PaCa-2	↓miR-27a, ↓cell growth, ↓invasion, ↑apoptosis, ↓onco-miR-223	[134, 135]
		AsPC-1, MIA PaCa-2	↓TGF-*β*1, ↓E-cadherin	[137]
	Colon cancer	HCT116	↓MMP-2, ↓FLT4, ↑G2/M cell cycle arrest	[140, 142, 143]
		HT-29, LoVo	↑Apoptosis, ↓NF-*κ*B, ↓Bcl-2, ↑Bax, ↓*β*-catenin	[141, 144]
		SW480, HCT116	↑G2/M cell cycle arrest, ↑p21waf1/cip1, ↑GADD45*α*, ↑ATM/p53, ↓cdc2, ↓cdc25A, ↓H3Ac, ↑Sfrp2, ↑Sfrp5, ↑Wnt5a	[145, 146]
		DLD-1, SW480, SW1116	↓EGF, ↑FOXO3, ↑p27Kip1	[148, 150, 151]
Urinary tract cancers	Kidney cancer	HK-2	↓PTH, ↑*α*-SMA, ↓CTGF, ↓mRNA, ↑E-cadherin	[153]
		A498, 786-O, Caki-2	↓miR-1260b, ↓DKK2, ↓Sfrp1, ↓Smad4	[154]
		A498, HEK-293, ACHN	↑2H3K4, ↑acetylated histones 3 and 4, RNA polymerase II, ↑3H3K4	[150, 155, 156]
	Bladder cancer	BDEC, TCCSUP	↑DNA damage, ↑cell growth, ↑cycle arrest of the G2/M phase, ↑apoptosis	[159–161]
		253J B-V	↓Growth of cells	[237]
	Prostate cancer	LAPC-4, LNCaP, PC-3	↓ER-*β*	[164–166]
		PC-3, DU145	↓miRNA-1260b, ↓Smad4, ↓Sfrp1	[167]
		C4-2B, ARCaP_M_, PC-3, PC-3-luc	↑Bax, ↓mCRPC growth	[169]
		DU145, PC-3	↑miR-34a, ↓HOTAIR, ↓miR-151	[170] [78
		C4-2B, LNCaP	↓Cell proliferation, ↑apoptosis	[171]
Genital cancers	Endometrial cancer	MES-SA, MES-SA-Dx5, SK-UT-1	↑DKK1, ↑p53, ↓Bax, ↓phospho-MEK, ↓*β*-catenin, ↓p27, ↑DNA fragmentation, ↑caspase-3	([179], [180])
		Ishikawa	↑DR5, ↑DR4, ↓caspase-8, ↓caspase-3, ↓PARP	[181]
		UtLM	↓TGF-*β*, ↓activin A, ↓Smad3	[183]
		ELT-3	↑PPAR*γ*	[187]
Bone cancer	Cervical cancer	HeLa	↑Apoptosis, ↑CHOP, ↑p-p70S6K1, ↑NF-*κ*B, ↑p-4E-BP1, ↑p-mTOR, ↑p-Akt, ↓TIMP-1, ↓survivin expression	[188, 191, 192, 196]
		TC-1	↑IFN-*γ*	[193]
		CaSki, HeLa	↓ERK1/2, ↓p38 MAPK	[194]
	Ovarian cancer	BG-1	pSmad3, ↓TGF-*β*, ↓EMT, ↓BPA, ↓E2, ↓TGF-*β*	[197]
		OCSLCs	↓FOXM1, ↓CD133, ↓ALDH1, ↓CD44	[198]
	Testicular cancer	TM4	↑Caspase-3, ↑necrosis, ↑apoptosis, ↑CPP32	[200, 201]
		MG-63	↑PPAR*γ*	[202]
	Osteosarcoma	MNNG/HOS	↑Akt, ↓NF-*κ*B	[203]
Skin cancer	Melanoma	C918	↓VE-cadherin mRNA, ↓Bcl-xL, ↓Bcl-2, ↑Apaf-1	[212–216]
		B164A5	↓Tumor weight, ↓volume, ↓quantity of melanin	[210]
		LiBr	↓Caspase-3, ↑apoptosis	[119]

*Abbreviations*: DNMT: DNA methyltransferase; TNF-*α*: tumor necrosis factor alpha; FADD: Fas-associated death domain; SF: sorafenib; 4-HPR: retinoid N-(4-hydroxyphenyl) retinamide; BIRC: baculovirus inhibitor of apoptosis repeat containing; AIF: apoptosis-inducing factor; VEGF: vascular endothelial growth factor; FGF2: fibroblast growth factor 2; SBDP: alpha spectrin to 145 kD spectrin breakdown product; CIP2A: cancerous prohibitor of protein phosphatase 2A; ABCC1: ATP-binding cassette subfamily C member 1; ABCG2: ATP-binding cassette superfamily G member 2; BCSCs: breast cancer stem cells; Bcl-2: B-cell lymphoma 2; HOTAIR: HOX transcript antisense intergenic RNA; miR-27a: microRNA-27a; HO-1: heme oxygenase-1; TSA: trichostatin A; ATRA: all-trans retinoic acid; Gli1: glioma-associated oncogene; siRNA: small-interfering RNA; miRNA: microRNA; Tyr15: phospho-cdc2; Ser 642: phospho-Wee1; PTEN: phosphatase and tensin homolog on chromosome 10; mRNA: messenger ribonucleic acid; MMP-9: matrix metallopeptidase-9; TPA: 12-*O*-tetradecanoylphorbol-13-acetate; JNK: c-Jun N-terminal kinase; ERK: extracellular signal-related kinase; MMP-2: matrix metalloproteinase-2; FLT4: Fms-related tyrosine kinase 4; H3Ac: histone H3 acetylation; FOXO: transcription factors of the forkhead box; CTGF: connective tissue growth factor gene; DKK1: Dickkopf-related protein 1; phospho-MEK: phosphorylated mitogen-stimulated protein kinase kinase; TIMP-1: tissue inhibitor of metalloproteinase-1; IFN-*γ*: interferon *γ*; FOX: forkhead box M1.

**Table 2:** The main key findings of clinical studies regarding genistein therapeutic potential.

Diseases	Key findings of clinical studies	Ref
Cancers	Anticancer and cytotoxic activity	
Breast cancer	Short-term dietary soy stimulated breast proliferation and progesterone receptor expression	McMichael-Phillips et al. [238]
	Weak estrogenic activity	Hargreaves et al. [239]
	Breast tissue modulation Biodistribution of the phytoestrogens in this tissue	Bolca et al. [243]
	↓Risk of fibroadenomas	[244]
	↓Efficacy, ↓prevention for breast cancer possible adverse effect in premenopausal women	[245]
Endometrial cancer	↓Endometrial hyperplasia without atypia, ↓endometrial thickness, ↑estrogen receptor beta (ER-*β*1) staining	[9]
Prostate cancer	↓Serum cholesterol No effects on biomarkers prostate-specific antigen (PSA) and p105erB-2	Urban et al. [250]
	↓Androgen-related biomarkers (KLK4), ↓cell cycle-related genes (p27Kip1)	[254]
	↑Apoptosis in low- to moderate-grade tumors	[256]
Urinary tract cancer	↑Superoxide generation after cisplatin treatment, ↓protein kinase	Yasuda et al. [265]
	↓Phosphorylated epidermal growth factor receptor (p-EGFR), ↓cell proliferation	Messing et al. [160]
Pancreatic cancer	↓Akt, ↓NF-*κ*B	El-Rayes et al. [266]
Colon cancer	No effect on colorectal epithelial cell proliferation	Adams et al. [268]
Menopause	↓Climacteric symptoms: ↓hot flushes, ↓visfatin, ↓insomnia, ↓depression	[269] [270].
Diabetes and lipid metabolism	↓Risk of diabetes	[280]
	↑Antioxidant effect on retinal cells in preretinopathic diabetes patients	[281]
	↑HDL, ↑apolipoprotein A-1, ↓LDL, ↓apolipoprotein B	[276]
Neurodegenerative diseases	Genistein did not show an improvement in cognition capacities in the elderly with Alzheimer's disease	[284]

## Abbreviations

*α*-SMA: Alpha-smooth muscle actin
ALT: Alanine transaminase
AST: Aspartate transaminase
ABCC1: ATP-binding cassette subfamily C member 1
AR: Androgen receptor
AIF: Apoptosis-inducing factor
ATRA: All-trans retinoic acid
AOM: Azoxymethane
BIRC: Baculovirus inhibitor of apoptosis repeat containing
Bcl-2: B-cell lymphoma 2/Bcl-2-associated X protein
BCSCs: Breast cancer stem cells
BW: Body weight
CYP1B1: Cytochrome P450 1B1
CIP2A: Cancerous prohibitor of protein phosphatase 2A
8-OHdG: 8-Hydroxy-2′-deoxyguanosine
Cdk: Cyclin-dependent kinase
COX-2: Cyclooxygenase-2
DMBA: 7,12-Dimethylbenz[a]anthracene
DFOG: 7-Difluoromethoxyl-5,4′-di-n-octylgenistein
DNMT: DNA methyltransferase
6-OHDA: 6-Hydroxydopamine
DCFH-DA: Dichloro-dihydro-fluorescein diacetate
dFMGEN: 7-Difluoromethyl-5,4′-dimethoxygenistein
DMH: 1,2-Dimethylhydrazine
DKK1: Dickkopf-related protein 1
DVL: Dishevelled protein
HDAC4/5/7: Histone deacetylase 4/5/7
EDCs: Endocrine-disrupting chemicals
NP: Nonylphenol
BPA: Bisphenol A
HCC: Hepatocellular carcinoma
EMT: Epithelial-mesenchymal transition
FAK: Focal adhesion kinase
H3Ac: Histone H3 acetylation
EGF: Epidermal growth factor
ERK: Extracellular signal-related kinase
FADD: Fas-associated death domain
FGF2: Fibroblast growth factor 2
FHF: Fulminant hepatic failure
5-FU: 5-Fluorouracil
FLT4: Fms-related tyrosine kinase 4
HO-1: Heme oxygenase-1
U937 cells: Human leukemia cells
Huh7.5: Male immortalized human hepatocarcinoma cells
HSCs: Male immortalized human hepatic stellate cells
HCPT: Hydroxycamptothecin
HUVECs: Human umbilical vein endothelial cells
BNL CL2: Human murine embryonic liver cells
HCT116 cells: Human colon cancer cells
I*κ*B: Inhibitory signaling pathways
Caco-2 cells: Intestinal colon cancer cells
LDH: Lactate dehydrogenase
IFN-*γ*: Interferon *γ*
ITT: Intratesticular testosterone
JNK: c-Jun N-terminal kinase
LC-MS: Liquid chromatography-mass spectrometry
MDA: Malondialdehyde
mRNA: Messenger RNA
TIMP-1: Tissue inhibitor of metalloproteinase-1
MAPK: Mitogen-activated protein kinase
MGO: Methylglyoxal
NA: Noradrenaline
NOR: Nucleolar organizer region
PES: Polyethersulfone
PVP: Polyvinylpyrrolidone
PARP: Poly(ADP-ribose) polymerase
PCNA: Proliferating cell nuclear antigen
PPAR*γ*: Peroxisome proliferator-activated receptor gamma
LNCaP: Prostate cancer cells
PXR: Pregnane X receptor
LC3 shRNA: Light chain 3 short hairpin RNA
PMA: Phorbol 12-myristate 13-acetate
PCa cells: Prostrate cancerous cells
ROS: Reactive oxygen species
RCC: Renal cell carcinoma
SACC-83 cell: Salivary adenoid cystic carcinoma-83 cell
SCLC cells: Small-cell lung cancer cells
SF: Sorafenib
SPI: Soy protein isolate
TNBC: Triple-negative breast cancer
TNF-*α*: Tumor necrosis factor alpha
TSA: Trichostatin A
TNFR-1: TNF receptor-1
TPA: 12-*O*-Tetradecanoylphorbol-13-acetate
Chk2: Serine/threonine-protein kinase 2
TCF: T cell factor
TLR2: Toll-like receptor 2
TRFIA: Time-resolved fluoroimmunoassay
UtLM: Uterine leiomyoma
VEGF: Vascular endothelial growth factor.
